# Targeting cancer signaling pathways and their therapeutic strategies

**DOI:** 10.1007/s12672-026-04708-8

**Published:** 2026-03-19

**Authors:** Maha M. Salem, Tarek M. Mohamed

**Affiliations:** Biochemistry Division, Chemistry Department, Faculty of Science, Tanta University, Tanta, Egypt

**Keywords:** Cancer, Angiogenesis, Mitochondrial dynamics and transfer, Metabolic regulation

## Abstract

Globally, cancer is still one of the leading causes of mortality because of its complicated metabolic reprogramming and angiogenesis, which underpin its development and progression in addition to its uncontrolled growth of cells. Tumor growth, invasion, and metastasis are all significantly impacted by the intricate and dynamic process of angiogenesis, which is managed by a variety of pro- and anti-angiogenic molecules. Furthermore, mitochondria, which are responsible for signal transduction and cellular energy synthesis, delicately control a variety of metabolic processes, influencing crucial biological phenomena including apoptosis and cell growth. Changes in mitochondrial dynamics, which stands for the fluctuating equilibrium between mito-fission and fusion and horizontal mitochondrial transfer (HMT) that is essential for preserving mitochondrial homeostasis and quality, are among the metabolic abnormalities commonly seen in tumor cells. Additionally, the malignant cells undergoes significant metabolic alterations that affect a variety of processes, including energy production, macromolecule biosynthesis, and nutrient uptake. Therefore, this review summarizes in general the different signaling pathways involved in tumor angiogenesis, reviews new findings about mitochondrial dynamics, and looks at key metabolic pathways such as OXPHOS, lipid metabolism, glycolysis, and glutaminolysis, with an emphasis on how these pathways contribute to the development of tumors. Also, examine further how tumor suppressors and oncogenes interact to modify these pathways. We also go over treatment strategies that target cancer angiogenesis, as well as changes in mitochondrial dynamics, transfer and metabolism.

## Introduction

**Fig. 1:**
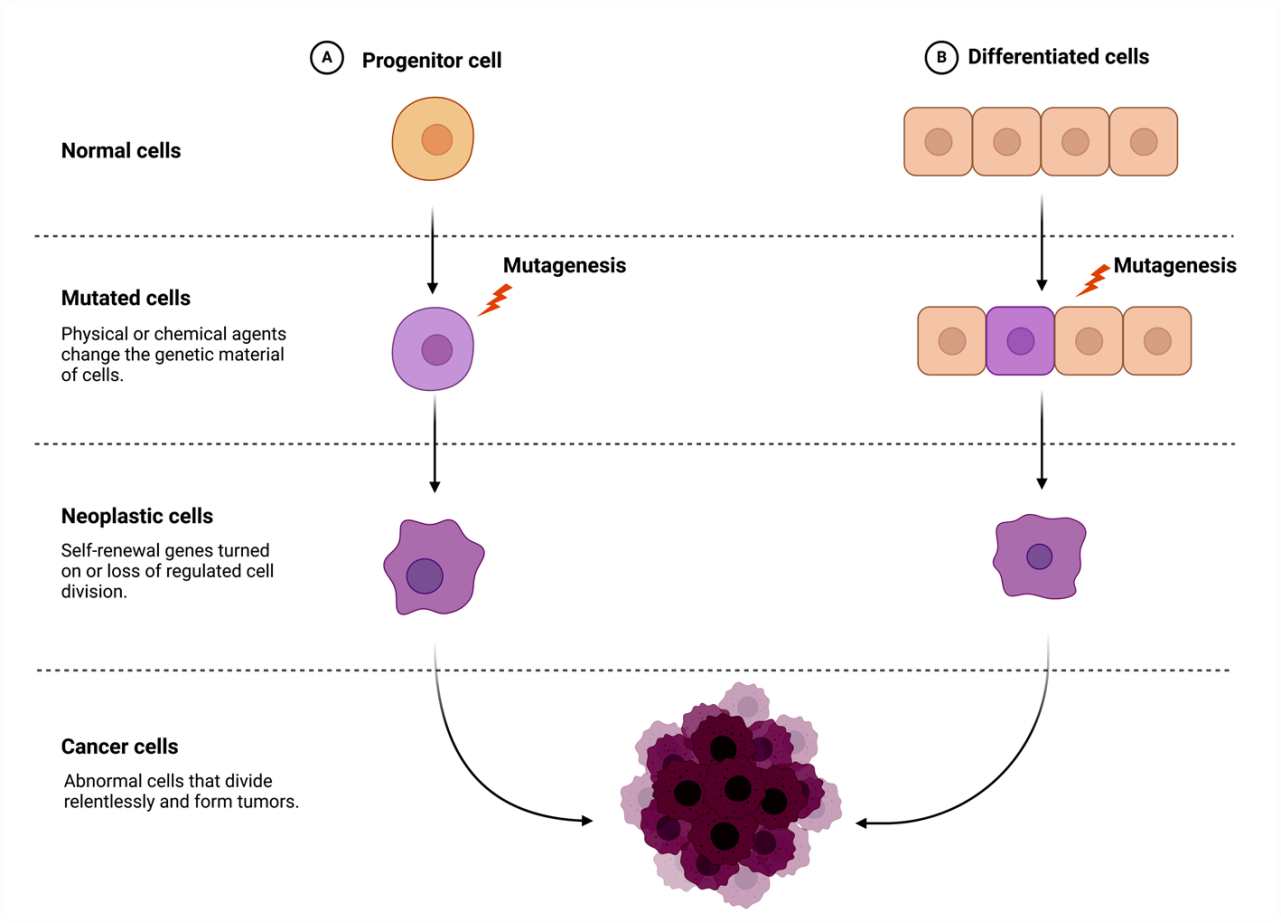
Cancer cell transformation

Cancer ranks as one of the most serious health challenges globally, holding the position of the second leading cause of mortality after heart disease. The World Health Organization indicated that each year, approximately 18 million individuals are diagnosed with cancer, and it is estimated that 10–15% of these cases will exhibit metastasis. The most common types of cancer include those affecting the breast, lung, colon, rectum, and prostate [1]. Cancer is a complicated disease characterized by uncontrolled cell proliferation and division, affecting numerous cellular and molecular pathways. Thereby, cancer is intended to circumvent the body's homeostatic systems and take advantage of this abnormal behavior for its growth and survival [2] (Fig. 1).

**Fig. 2:**
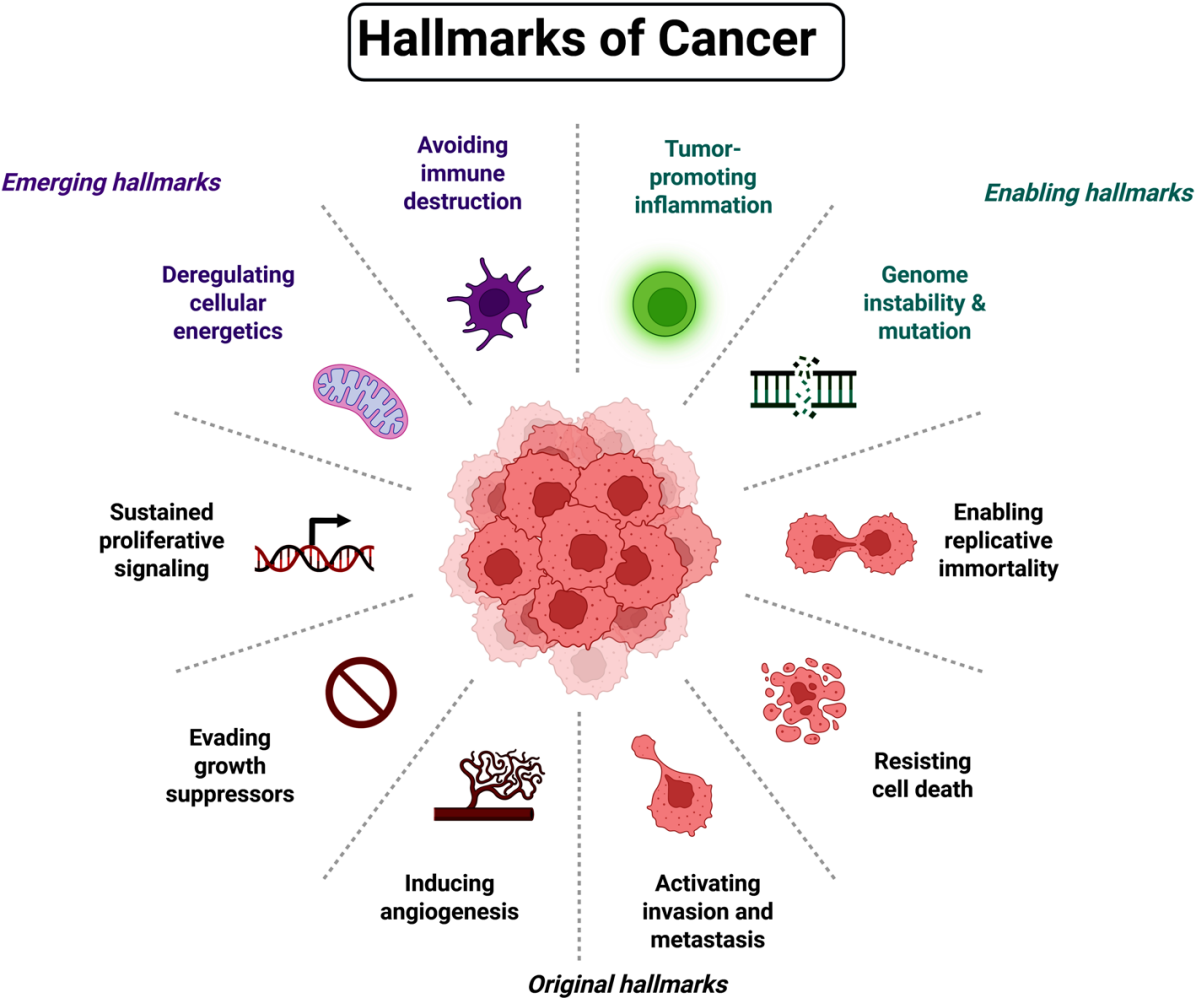
Hallmarks of cancer

Cancer frequently results from the combination of environmental exposures and genetic predispositions, which lead to the change of important regulatory genes. The hallmarks of cancer cells thus develop apoptosis resistance, prolonged proliferative signaling, invasion and metastasis activation, growth suppressor evasion, and angiogenesis induction, all of which are essential for tumor progression [3] (Fig. 2).

**Fig. 3:**
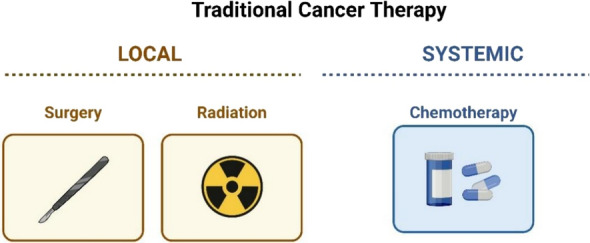
Traditional cancer treatment methods

Furthermore, the initial tumor may respond to therapeutic measures such as local surgery or radiation therapy. But tumor metastasis shows systemic propensity, whereas treatment requires a combination of immunotherapy, targeted treatments, and chemotherapy (Fig. 3). Despite chemotherapy being the most popular treatment, it has several undesirable side effects, such as indiscriminately destroying healthy cells and causing chemotherapeutic resistance [4].

Therefore, new targets and early biomarkers must be urgently developed by understanding intricate signaling cascades and biological mechanisms to improve the efficacy of cancer treatments and increase patient survival rates. Researchers aspire to disrupt the growth of cancer cells via focusing on all signaling pathways that are altered as cell division, apoptosis, and metabolism to improve treatment outcomes uniquely.

**Fig. 4:**
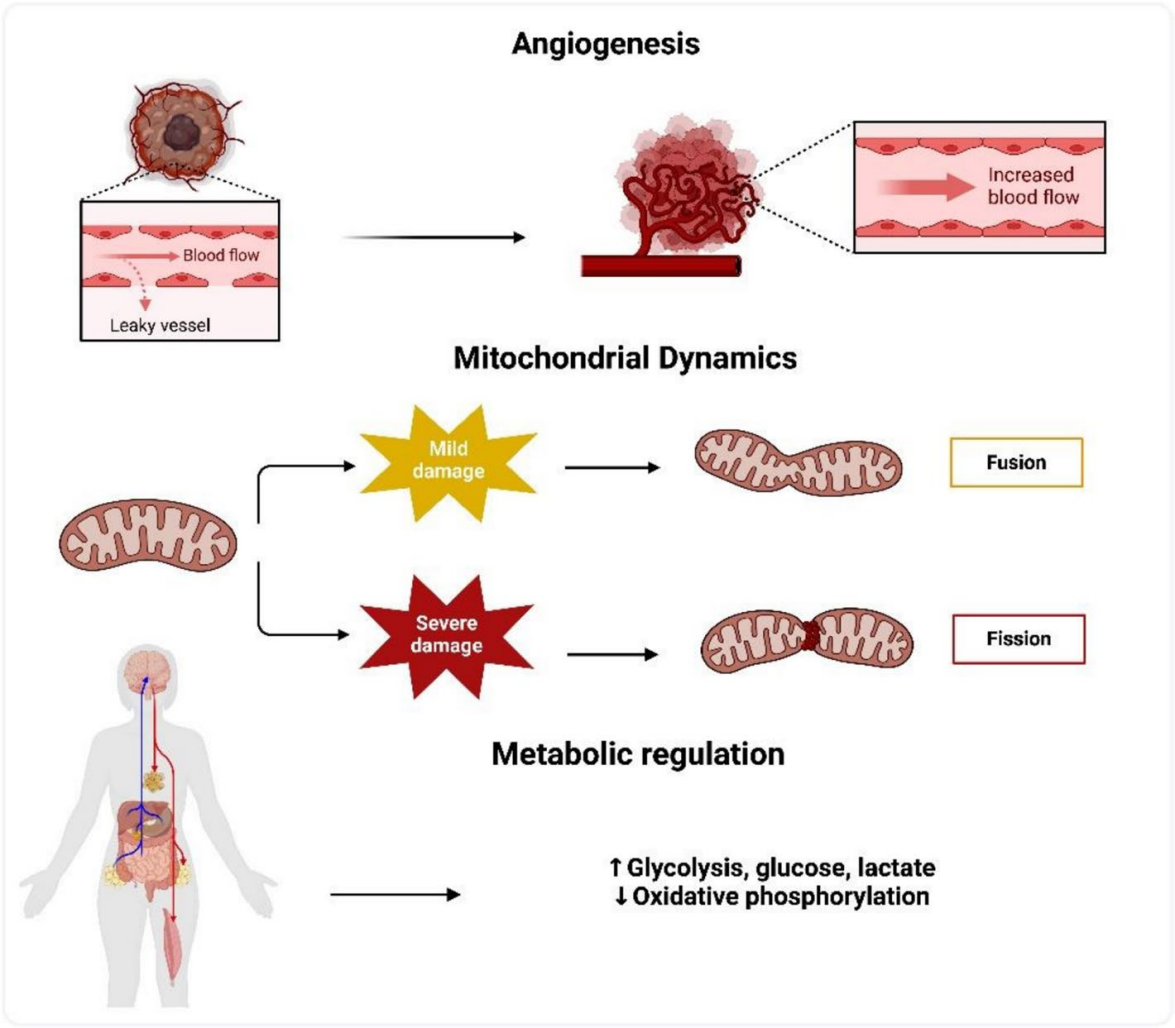
Three essential domains pertinent to cancer progression and treatment

In this review, we will focus on the three key areas of angiogenesis, mitochondrial dynamics, horizontal mitochondrial transfer and metabolic regulation that are relevant to the development and management of cancer. Angiogenesis provides cancer cells with vital nutrients and oxygen, which is crucial for cancer development and spread. The metabolism of cancer cells and their resistance to therapy depend heavily on mitochondrial dynamics and transfer, which include processes like fusion and fission. Cancer cells may multiply quickly and survive in a variety of environments due to metabolic control, particularly the reorganization of energy generation pathways (Fig. 4).

## Angiogenesis and therapeutic strategies

### Mechanism of tumoral angiogenesis

Angiogenesis is the process by which preexisting capillaries give rise to new blood vessels, which ultimately form a mature, consistent, and comprehensive vascular system. This process involves the breaking down of the basement membrane as well as the activation, development, and migration of endothelial cells (ECs), which are regulated by a range of pro- and anti-angiogenic substances [5].

**Fig. 5:**
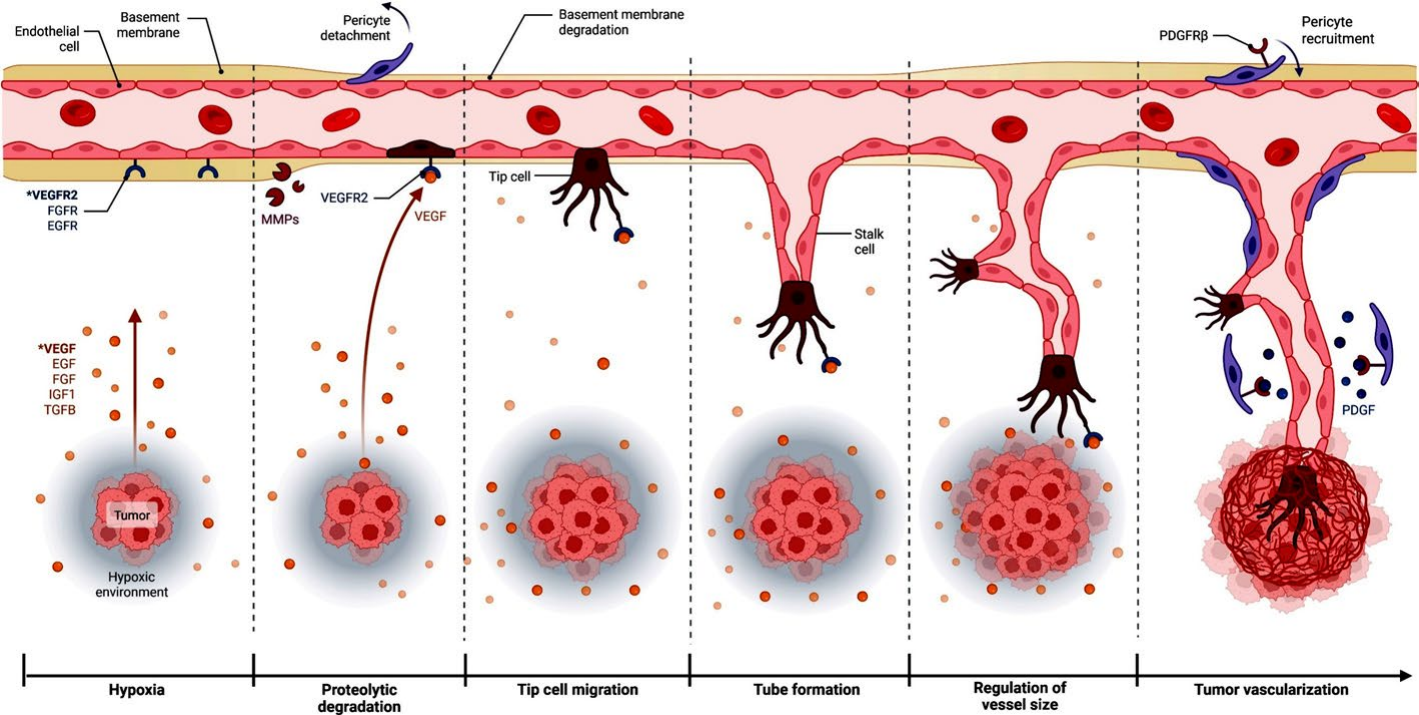
The progression of the cancer cell towards angiogenesis

Additionally, angiogenesis has a role in the development of several malignant tumors, including renal cell carcinoma (RCC), colorectal cancer (CRC), breast cancer (BC), melanoma, and non-small cell lung cancer (NSCLC). The tumor is a biological tissue that requires oxygen and nutrients significantly more than healthy tissue cells because of its fast growth, high metabolism, and persistent vitality. An avascular state is the first stage of tumor growth, during which the tumor has not become aggressive and diffuses oxygen and nutrients into the surrounding tissue. Consequently, intratumoral vascularization is uncommon because of the low amounts of pro-angiogenic molecules and signals that block blood vessels in the extracellular matrix, which lock or limit tumor angiogenesis to a quiescent state [6] (Fig. 5).

When tumor cells grow quickly, a microenvironment characterized by higher interstitial pressure, acidity, and more severe hypoxia formed in organizations distant from the tumor tissue's blood arteries, which encouraged the tumor tissue to proliferate and invasion (Fig. 5). To sustain an extremely active angiogenesis stage, tumor cells continuously release or upregulate a variety of pro-angiogenic factors to activate endothelial cells with proliferative potential (called “Tip-cells”) are recruited and will initiate the appearance of angiogenic buds in local vessels. The “budding” process is aided by the degradation of the endothelial basement membrane. This leads to remodel of the extracellular matrix, differentiation of endothelial cells, and the detachment of pericytes which ultimately causes tumor proliferation, diffusion, and metastasis [7].

### Signaling pathways in tumor angiogenesis

#### Vascular endothelial growth factor (VEGF)

**Fig. 6:**
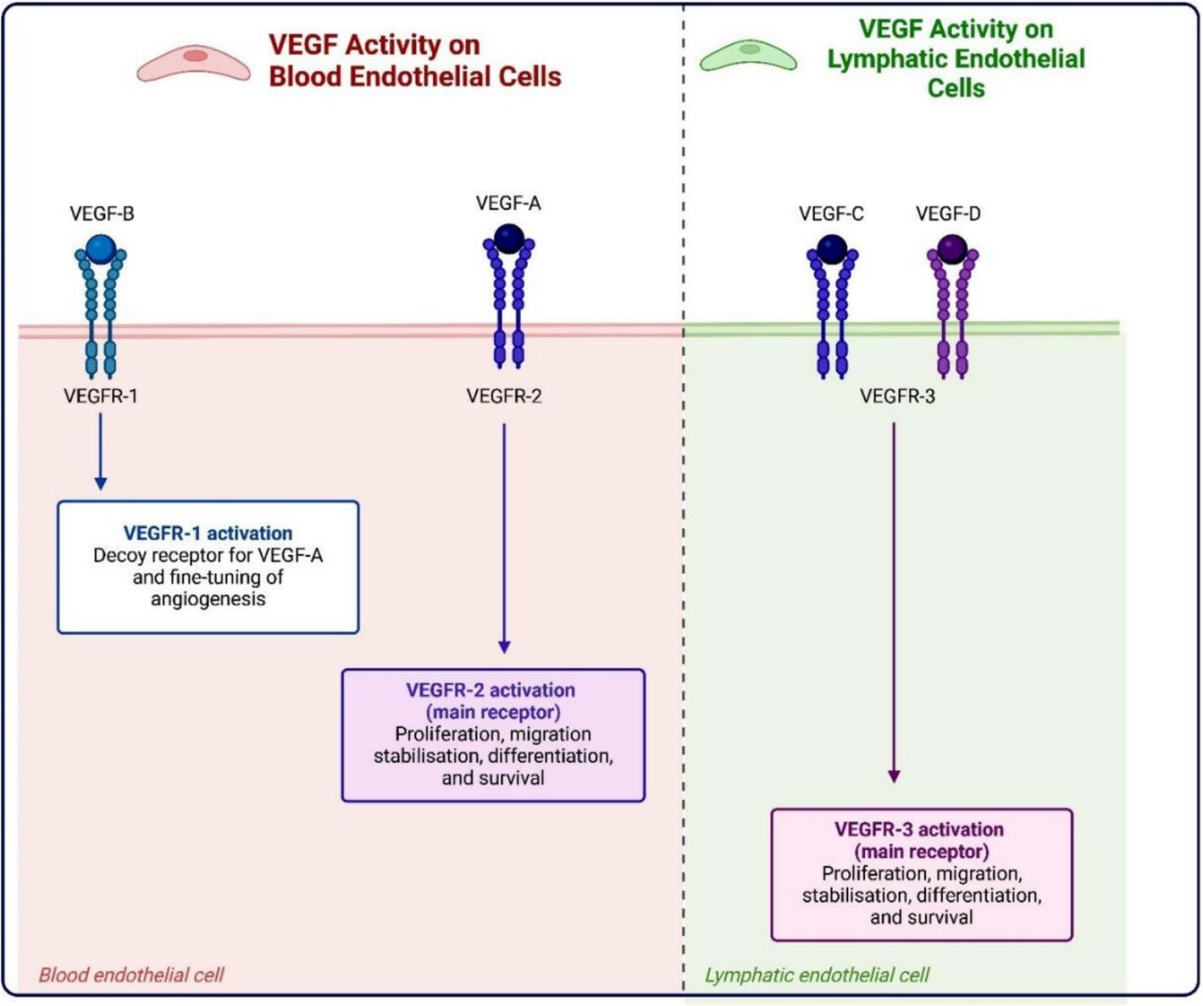
VEGFRs are the tyrosine kinase receptors for vascular endothelial growth factors

VEGF is the prototypical member of a growth factor family of five structurally analogous molecules: VEGF, placental growth factor (PlGF), VEGFB, VEGFC, and VEGFD. The biological functions of these angiogenic factors are predominantly mediated by two tyrosine kinase receptors (TKRs), vascular endothelial growth factor receptor 1 (VEGFR1) and VEGFR2, primarily expressed in endothelial cells, although the non-TKR family of neuropilins (NRPs) also transmits certain VEGF functions. Members of the VEGF family demonstrate selective affinity for VEGFRs: VEGF associates with VEGFR1 and VEGFR2; PlGF and VEGFB preferentially engage with VEGFR1; and VEGFC and VEGFD serve as natural ligands for VEGFR3 but can also activate VEGFR2 post-proteolysis (Fig. 6). VEGFR2 is the primary mediator of the vascular functions of the VEGF family, while VEGFR1 appears to function as a decoy receptor in some contexts, due to its weak intrinsic signaling and strong VEGF binding affinity, which inhibits VEGF from associating with VEGFR2. VEGFR3 is predominantly expressed in lymphatic endothelial cells and transmits lymphangiogenic signals for VEGFC and VEGFD, however it is also temporarily expressed in angiogenic endothelial cells during the development of sprouting tips (Fig. 6) [8–10].

VEGF activates multiple intracellular signaling pathways that are implicated in angiogenesis and inflammation. The regulation of VEGF expression involves various cytokines, including IL-1, IL-6, and nitric oxide, as well as growth factors such as epidermal growth factor (EGF), platelet-derived growth factor (PDGF), and tumor necrosis factor alpha (TNF-α). Additionally, hormones like thyrotropin, steroid hormones, insulin, and corticotropin play a role in this regulation [11]. VEGF expression is stimulated by the loss of tumor suppressor genes, such as p53, and the activation of oncogenes, including ras, v-src, and Her2. The primary factor driving the overexpression of VEGF remains hypoxia, which initiates the stabilization of the alpha subunit of HIF-1 [12]. The overexpression of VEGF correlates significantly with metastatic dissemination and lymphatic involvement. Clinically, patients exhibiting elevated VEGF levels demonstrate poorer survival rates compared to those with low or absent levels. Preoperative levels of VEGF are associated with more advanced stages of cancer. Levels of VEGF have been proposed as predictors of metastatic potential and identified as an independent risk factor for nodal status and adjuvant chemotherapy [13].

**Fig. 7:**
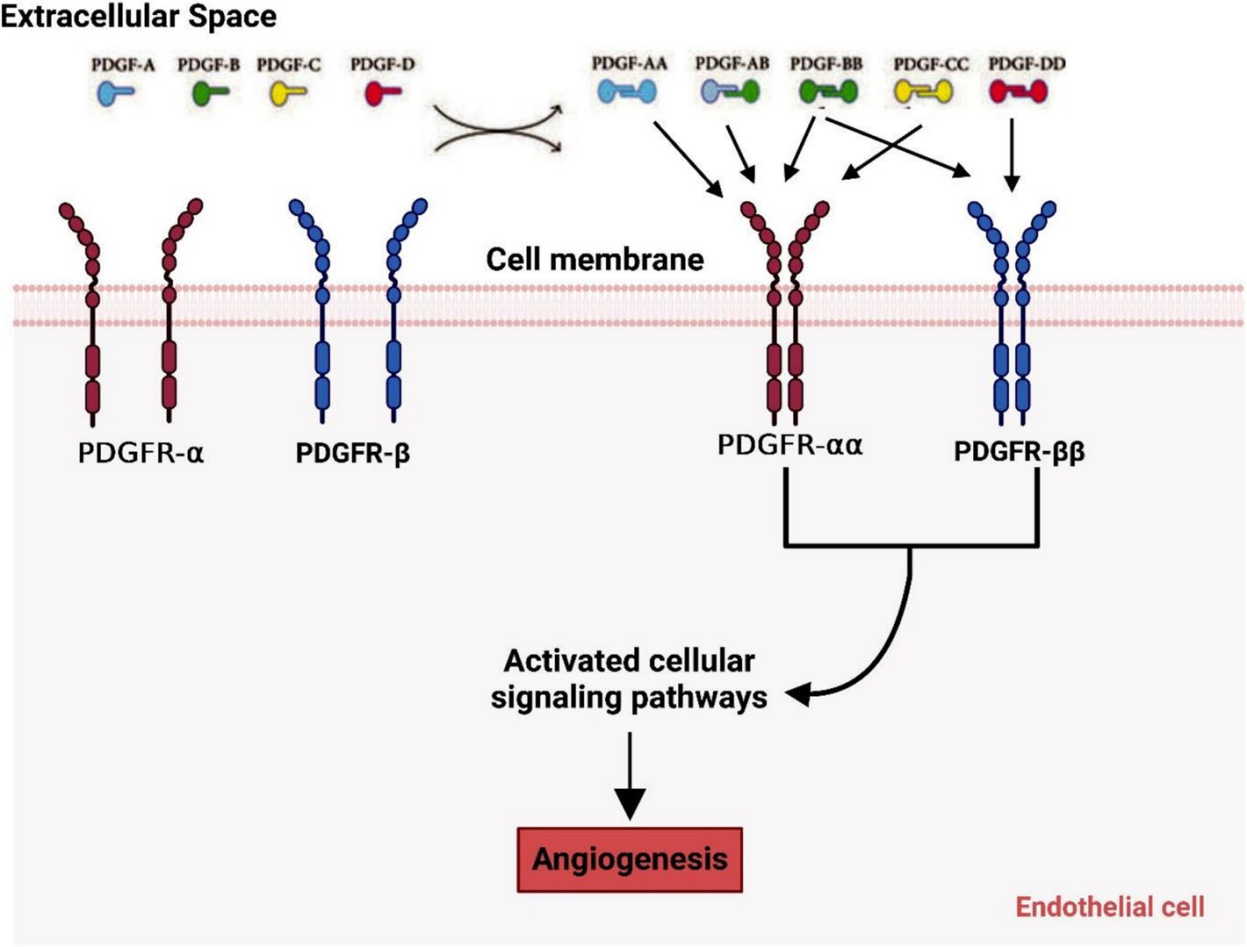
Platelet-derived growth factor and its receptors in inducing angiogenesis

Further, autophosphorylation and signal transduction of VEGFR-2/VEGF-A can strongly activate common signaling pathways linked to angiogenesis and the growth and survival of endothelial cells, including PI3K/AKT/mTOR, MAPK, and Ras/Raf/MEK/ERK (Fig. 7) [14]. Research focusing on the inhibition of the VEGF/VEGFR signaling pathway has emerged as a primary target in the development of novel antiangiogenic drugs, leading to the creation and approval of various therapeutic agents.[13].

#### Platelet-derived growth factor (PDGF/PDGFRs)

The PDGF is a substance that platelets and certain stromal cells secrete and that aids in angiogenesis or coagulation. Angiogenesis, wound healing, cell growth, and differentiation are all impacted by PDGF. PDGFs consist of 4 soluble, deactivated polypeptide chains: PDGF-A, PDGF-B, PDGF-C, and PDGF-D. These chains are converted into active homodimers or heterodimers: PDGF-AA, PDGF-AB, PDGF-BB, PDGF-CC, and PDGF-DD, which then carry out biological functions [15]. Cell differentiation, metastasis, invasion, proliferation, and angiogenesis are all fueled by PDGF-AA, which functions as a cancer promoter through PDGFR-α. PDGF-DD/PDGFR-β can aggravate cancer growth and metastasis (Fig. 7) [16]. PDGF-BB is among the most researched components of the PDGF family due to its strong ability to drive cancer through a variety of downstream signaling pathways (including the PI3K/AKT and MAPK pathways) (Fig. 9). It controls the migration and proliferation of PDGF-dependent cells [17]. Research has demonstrated that PDGFs and PDGFR-α/β are excessively active in a variety of cancerous tissues, such as hepatocellular carcinoma (HCC), ovarian cancer, and breast cancer [18]. PDGFRs can be neutralized or inhibited to prevent the growth, invasion, metastasis, and angiogenesis of carcinomas.

#### Epidermal growth factor (EGF/EGFRs)

EGF plays a major role in tumor angiogenesis, adhesion, apoptosis, migration, differentiation, proliferation, and growth of cells through binding to its receptor, EGFR. EGFR mediates downstream signaling pathways (MAPK, PI3K/AKT), which enhance endothelial cell proliferation and differentiation (Fig. 9) [19]. Additionally, the binding of EGF with EGFR encourages mitosis and overexpressed a variety of angiogenic factors, including VEGF, which indirectly triggers tumor angiogenesis. EGFR is typically activated by many malignant tumors, which mediate genes expression to directly stimulate tumor development and angiogenesis that result in tumor invasion and metastasis [20].

#### Fibroblast growth factor (FGF/FGFRs)

**Fig. 8:**
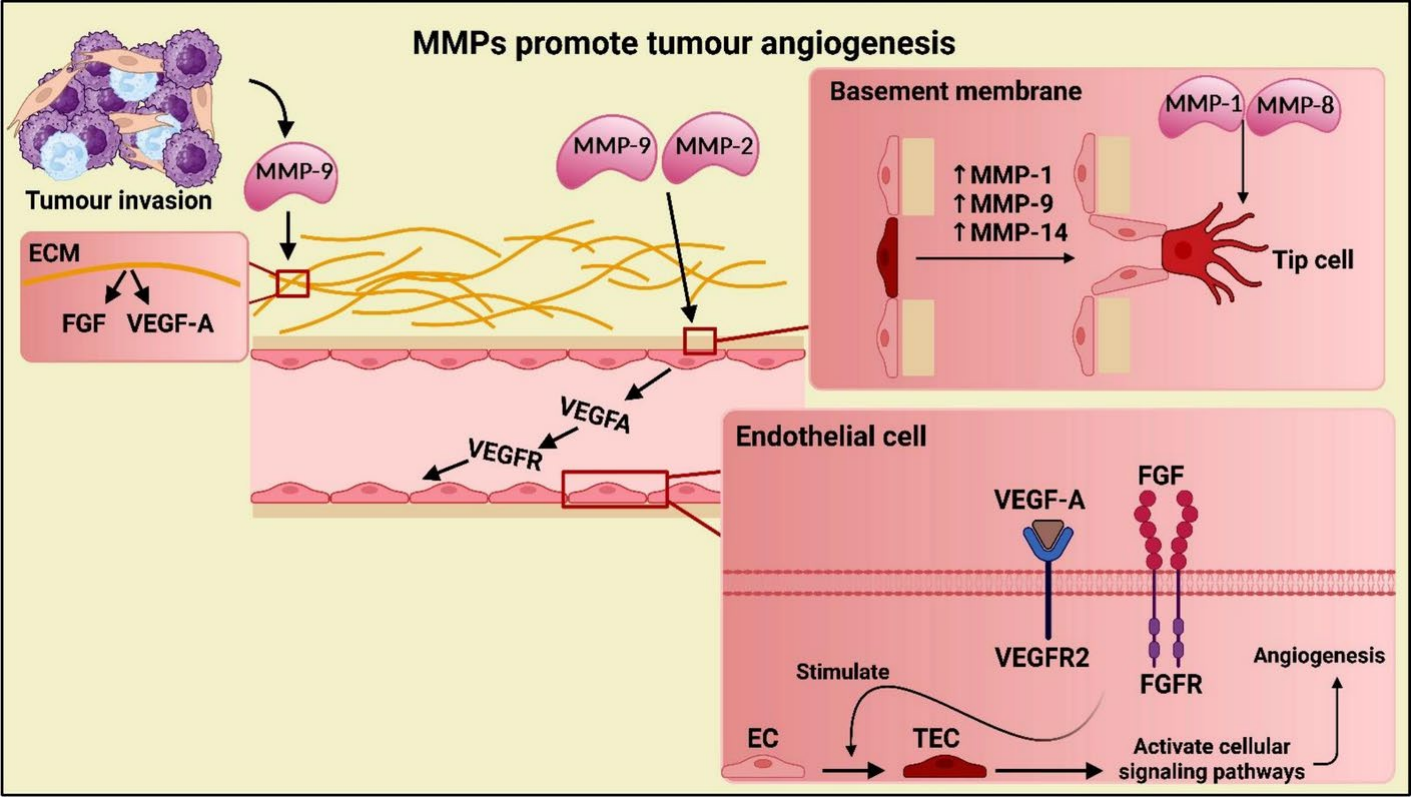
Matrix metalloproteinases’ (MMPs) role in inducing angiogenesis

**Fig. 9:**
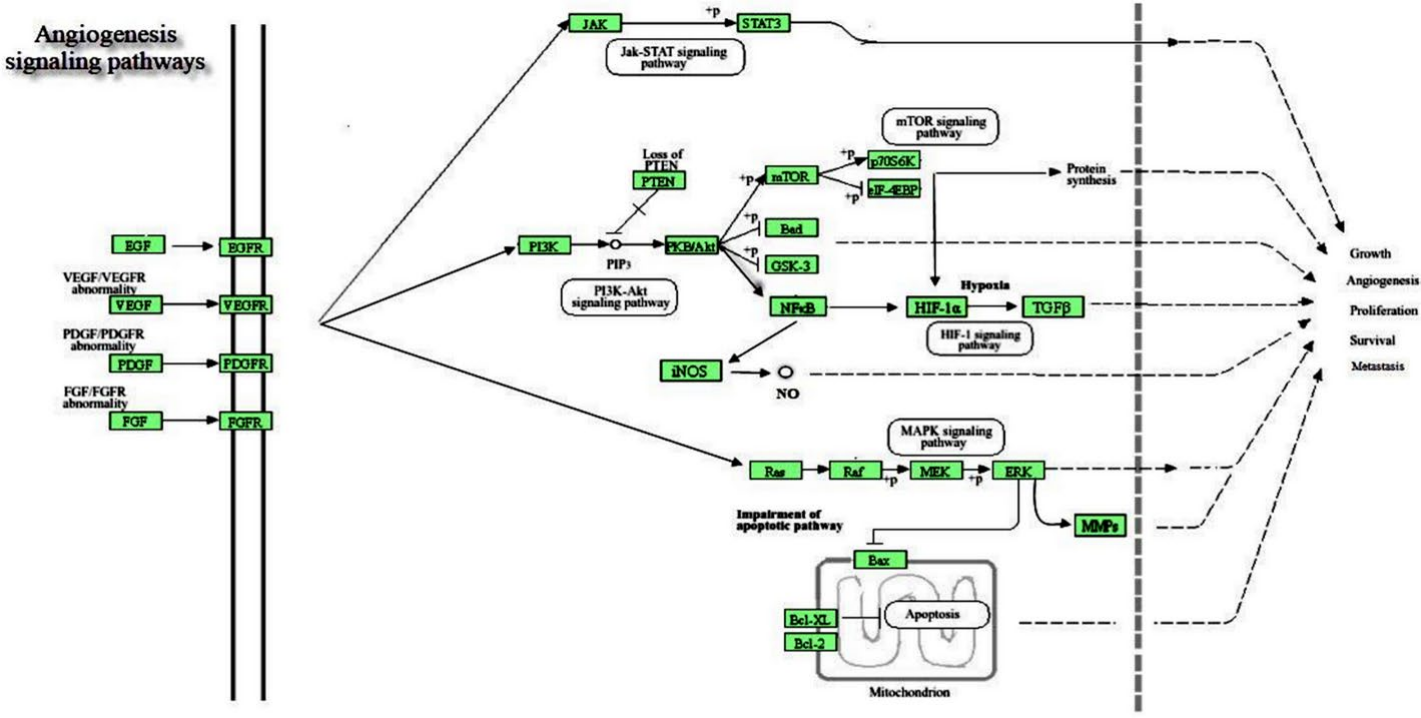
Diagrammatic representation of how cellular signaling pathways interact during tumor angiogenesis

FGF controls angiogenesis, wound healing, tissue homeostasis, and the development of cancer. Five members of the FGFR1–5 transmembrane receptor family mediate angiogenesis, drug resistance, migration, and survival in cancer cells via activating the PI3K/AKT and Ras/Raf-MAPK signaling pathways and auto-phosphorylating them (Fig. 9) [21]. FGF/FGFR signaling is essential for tumor angiogenesis because it stimulates the release of matrix metalloproteases (MMPs) (Fig. 8) and controls endothelial cell differentiation, migration, proliferation, morphological alterations, and vascular maturation [22]. In anti-VEGFR therapy, aberrant FGF/FGFR activations are crucial alternative angiogenic mechanisms that lead to drug resistance.

#### Transforming growth factor-β (TGF-β)

TGF-β is a cytokine that is released and implicated in tumor angiogenesis, apoptosis, cell development, differentiation, proliferation, inflammation, and immunological responses in addition to body homeostasis [23]. During the initial phases of cancer development, TGF-β functions as a tumor suppressor by primarily suppressing cell proliferation and modifying immune responses to sustain cellular homeostasis and prevent the growth and dissemination of pre-cancerous cells. Dysregulation of TGF-β signaling in the early stages contributes to the onset and progression of many malignancies. The tumor-suppressive actions of TGF-β were facilitated by two principal transcriptional pathways. TGF-β first increases the production of cyclin-dependent kinase (CDK) inhibitors, such as p15, p21, and p27, which impede CDK function by obstructing cell cycle progression. Secondly, TGF-β suppresses C-MYC expression, a proto-oncogene that facilitates cellular proliferation [24]. The MUC1 transmembrane glycoprotein is aberrantly expressed in several epithelial malignancies and influences multiple signaling pathways upon its overexpression. The high levels of MUC1 protein activate TGF-β, NF-κβ and β-catenin and JNK pathways. This in return enhances the stability and viability of cells by activating C-MYC in tumor suppressive effects during early stages of development [25]. TGF-β also promotes apoptosis in various cell types as an additional mechanism to prevent tumor growth. The pro-apoptotic impact is facilitated by both SMAD-dependent and SMAD-independent mechanisms. The SMAD-dependent pathway induces the phosphorylation and activation of receptor-regulated SMAD proteins (SMAD2 and SMAD3), leading to the creation of a complex through the binding of common-mediator SMAD (SMAD4). This complex translocated to the nucleus, modulating gene expression associated with apoptosis and cell cycle arrest [26]. The SMAD-independent route is thought to engage alternate signaling molecules and pathways that also play a role in the pro-apoptotic and anti-proliferative effects of TGF-β signaling. The abrogation of TGF-β’s tumor-suppressive properties may lead to increased cell proliferation, apoptosis, and metastasis, hence promoting the progression from early-stage tumours to more aggressive and advanced cancer types. Comprehending the molecular basis of TGF-β's tumor-suppressive roles in early-stage malignancies is crucial for formulating targeted therapeutics and preventing cancer progression [27].

#### Hypoxia-inducible factor-1(HIF-1)

The most prevalent characteristic of the tumor microenvironment is hypoxia, which is invariably linked to tumor angiogenesis, aggressiveness, and recurrence. Hypoxia-inducible factor-1 is a transcription factor that controls energy metabolism, cell tolerance to hypoxia, cell survival, adhesion, migration, and cell death [28]. In hypoxic situations, HIF-1α and HIF-1β dimerize by nuclear translocation, which is influenced by oxygen concentration. This, in turn, activates several target genes as VEGF, TGF-β, PDGF, and MMPs. The intricate procedure stimulates tumor angiogenesis and increases the invasiveness and affinity of tumor cells for survival (Fig. 9) [29].

#### Nuclear factor κβ (NF-κβ)

One of the body's key transcription factors is the nuclear factor κβ (NF-κβ), which has a role in angiogenesis, oxidative damage, inflammation, immunological responses, and cell survival (Fig. 9) [30]. By altering the level of angiogenic factor expression, particularly VEGF/ NF-κβ controls the growth of different types of carcinomas [31].

#### Matrix metalloproteinases (MMPs)

MMPs are calcium and zinc-dependent endopeptidases that are released by stromal and connective tissue cells, including neutrophils, fibroblasts, ECs, macrophages, osteoblasts, and lymphocytes [32]. MMPs are key enzymes that have a role in remodeling the basement membrane and the destruction of extracellular matrix (ECM) in different types of angiogenesis. This remodeling enzymatically breaks down the peptide bonds of elastin, collagen, fibronectin, and laminin (Fig. 8) [33].

The angiogenic system is made up of a variety of biomolecules that either promote or inhibit angiogenesis, such as growth factors (fibroblast growth factor, vascular endothelial growth factor, and transforming growth factor), proteases (matrix metalloproteinase), protein kinase B (AKT), signaling molecules mechanistic target of rapamycin (mTOR), transcription factors (nuclear factor and hypoxia-inducible factor), mitogen-activated protein kinases (MAPKs), were serine/threonine kinases that function in many cellular responses to stimuli such as mitogens, osmotic stress, heat shock, and proinflammatory cytokines. MAPKs participate in numerous biological processes, including proliferation, gene expression, differentiation, mitosis, cell survival, and apoptosis. Mammals have four principal MAPKs: (1) ERK1/2, (2) c-Jun N-terminal kinase (JNK)1–3, (3) p38, and (4) ERK5. Alongside these four principal MAPKs, many atypical MAPKs (such as ERK 3/4, ERK 7/8, and Nemo-like kinase [NLK]) have been discovered, exhibiting less clearly defined functions and distinct activation methods [34]. Abnormalities in MAPK signaling were pivotal in the development and progression of cancer. Notch signaling functions as both an oncogenic agent and a tumor suppressor across different cancer types [35].

Dysregulation of this system facilitates epithelial-mesenchymal transition and angiogenesis in malignancies, which were intricately associated with cancer proliferation, invasion, and metastasis. The Notch signaling pathway also plays a role in preserving stem-like characteristics in cancer cells, hence increasing cancer invasiveness. The regulatory role of the Notch signaling pathway in cancer metabolic reprogramming and the tumor microenvironment suggests its pivotal involvement in balancing oncogenic and tumor suppressive effects [36].

All these biomolecules initiate signaling pathway transduction via transmembrane receptors that promote gene expression endothelial cell survival, proliferation, and angiogenesis (Fig. 9).

### Therapeutic strategies using angiogenic inhibitors

Anti-angiogenic therapy is accomplished by limiting the blood supply to tumor tissue, thus preventing tumor growth and metastasis. Despite the reality that mechanistic and molecular research have shown many regulators are involved in tumor angiogenesis, the exploration of the angiogenic inhibitors continues, especially focusing on growth factors and their receptor signalling pathway because of their dominance in the angiogenic system [37, 38].

#### Endogenous direct inhibitors of angiogenesis

Additionally, there exist endogenous inhibitors of angiogenesis that disrupt pro-angiogenic factors during the aforementioned stages of angiogenesis illustrated in Fig. 5. These endogenous inhibitors specifically target endothelial cells in the sprouting arteries, inhibiting their proliferation and migration, hence blocking stimulation by proangiogenic stimuli. Many endogenous inhibitors have been found, including angiostatin, endostatin, β-arrestin, thrombospondin 1 and 2, endorepellin, fibulin, canstatin, and tumstatin, which are released during the proteolysis of the extracellular matrix [39]. These factors impede endothelial cell proliferation and migration in reaction to several pro-angiogenic stimuli, including VEGF, bFGF, IL-8, and PDGF. Moreover, endogenous inhibitors originate from several cells, including IFN-α (interferon-α), multiple interleukins excluding IL-8 (specifically IL-1β, IL-4, IL-12, and IL-18), as well as other factors such as tissue inhibitors of metalloproteinases, and 2-methoxyestradiol. The list continues to expand with the continual emergence of novel factors, such as isthmin 1 and multimerin-2 [40]. Consequently, these endogenous direct angiogenic inhibitors are essential for preserving angiogenic equilibrium and can influence the rate of neovascularization in both health and sickness. The strategy of augmenting endogenous angiogenic inhibitors may be regarded as a rather safe long-term anticancer treatment.

#### Exogenous indirect angiogenic inhibitors

**Fig. 10:**
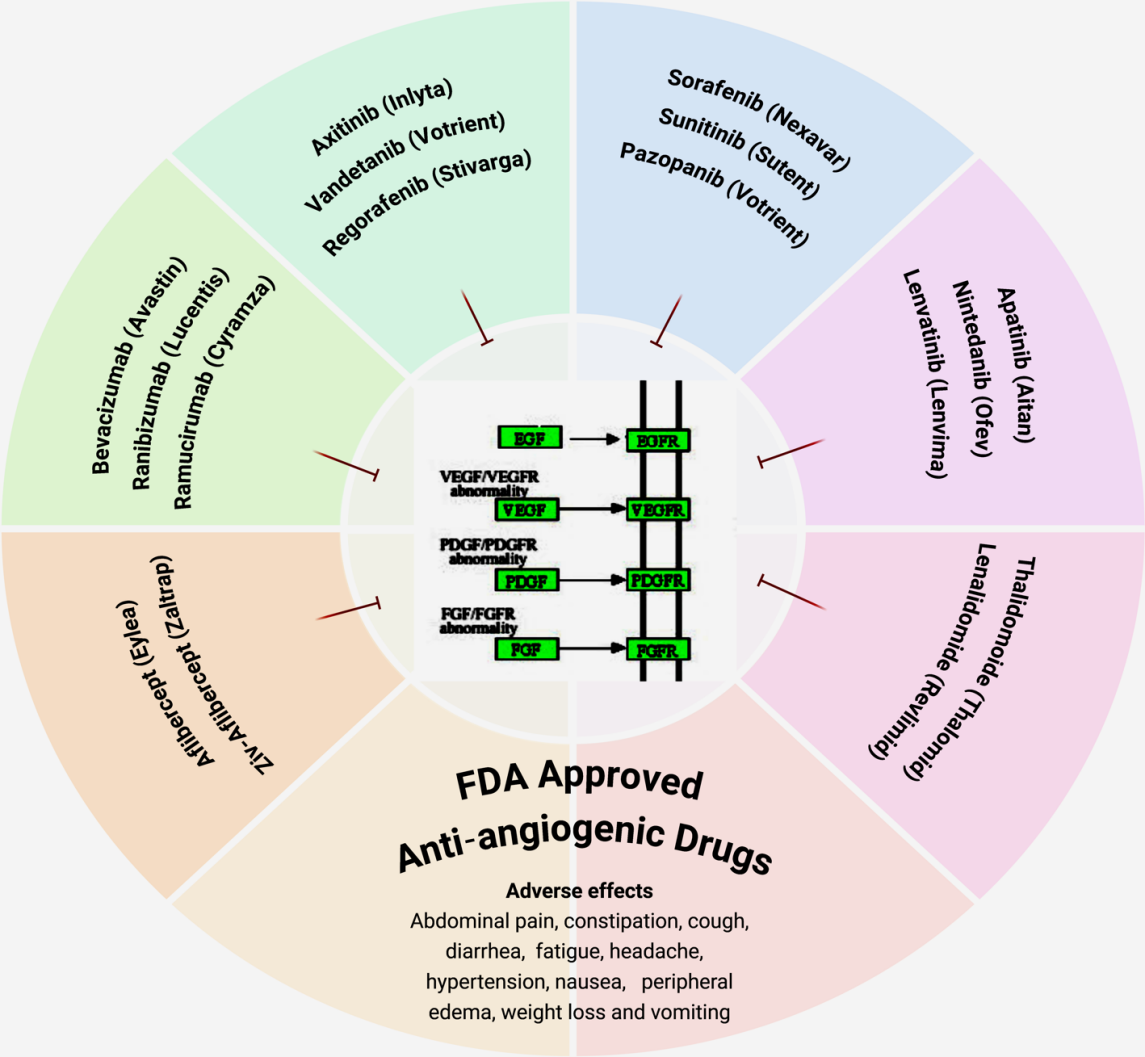
Therapeutic strategies using FDA anti-angiogenic inhibitor drugs

Drugs that indirectly restrict angiogenesis by targeting cancer cells or related stromal cells can impede the development of new blood vessels, so halting tumor growth without totally eradicating it. These indirect inhibitors can diminish the expression or function of pro-angiogenic molecules such as VEGF or EGFR. Nonetheless, monotherapies targeting angiogenesis have not achieved the anticipated efficacy; hence, combination treatments with traditional chemotherapeutic agents are favored instead. Chemotherapeutic agents, like as paclitaxel and cyclophosphamide, exhibit significant anti-angiogenic properties, primarily by disrupting the cytoskeleton and migration of endothelial cells in neoplastic vasculature. Gefitinib (ZD1839, Iressa®) is a small molecular weight EGFR tyrosine kinase inhibitor (TKI) that has demonstrated anti-angiogenic properties in colon (SW480, CaCo2), breast (ZR-75–1, MCF-7), ovarian (OVCAR-3), and gastric (KATO III, N87) cancers. These cells co-express TGF-α and EGFR as pro-angiogenic factors [41]. Tyrosine kinase inhibitors are a rapidly expanding class of anti-angiogenic agents that target one or more pro-angiogenic receptors, including VEGFR, EGFR, FGFR, and PDGFR. New tyrosine kinase inhibitors (TKIs) are continually incorporated into the category, such as CM082 (a VEGFR inhibitor) [42] and DW14383 (a pan-FGFR inhibitor) [43]. A separate category of anti-angiogenic agents comprises monoclonal antibodies targeting pro-angiogenic factors. Bevacizumab (Avastin®) is a recombinant humanized monoclonal antibody targeting VEGF-A, resulting in the deprivation and subsequent growth suppression of cancer cells [44]. This medicine is frequently administered alongside other chemotherapeutics to enhance efficacy, including irinotecan, leucovorin, fluorouracil, carboplatin, paclitaxel, or platinum-based chemotherapy. Additional comparable instances encompass ramucirumab (Cyramza®) [45], panitumumab (Vectibix®) [46], and cetuximab (Erbitux®) [47]. Ultimately, there are medicines that focus on additional angiogenesis-related factors, including MMPs and Hsp90 (heat shock protein 90), as well as tumor-associated populations including stromal cells and bone marrow-derived myeloid cells Fig. 10.

## Mitochondrial dynamics and signaling pathways

The "powerhouse of the cell” is the mitochondria, which represents the main location for aerobic respiration and the energy-producing cellular compartment. In addition to their primary bioenergy role, mitochondria also supply the elements needed for tumor anabolism, tumor cell redox, and cell death regulation [48].

Mitochondrial fusion and fission are processes known as mitochondrial dynamics that control the number, shape, and quality of mitochondria [49]. Cell migration, metabolism, and proliferation are all strongly related to mitochondrial processes, and they are all strictly controlled by different proteins [50].

### Mitochondrial fission and fusion

**Fig. 11:**
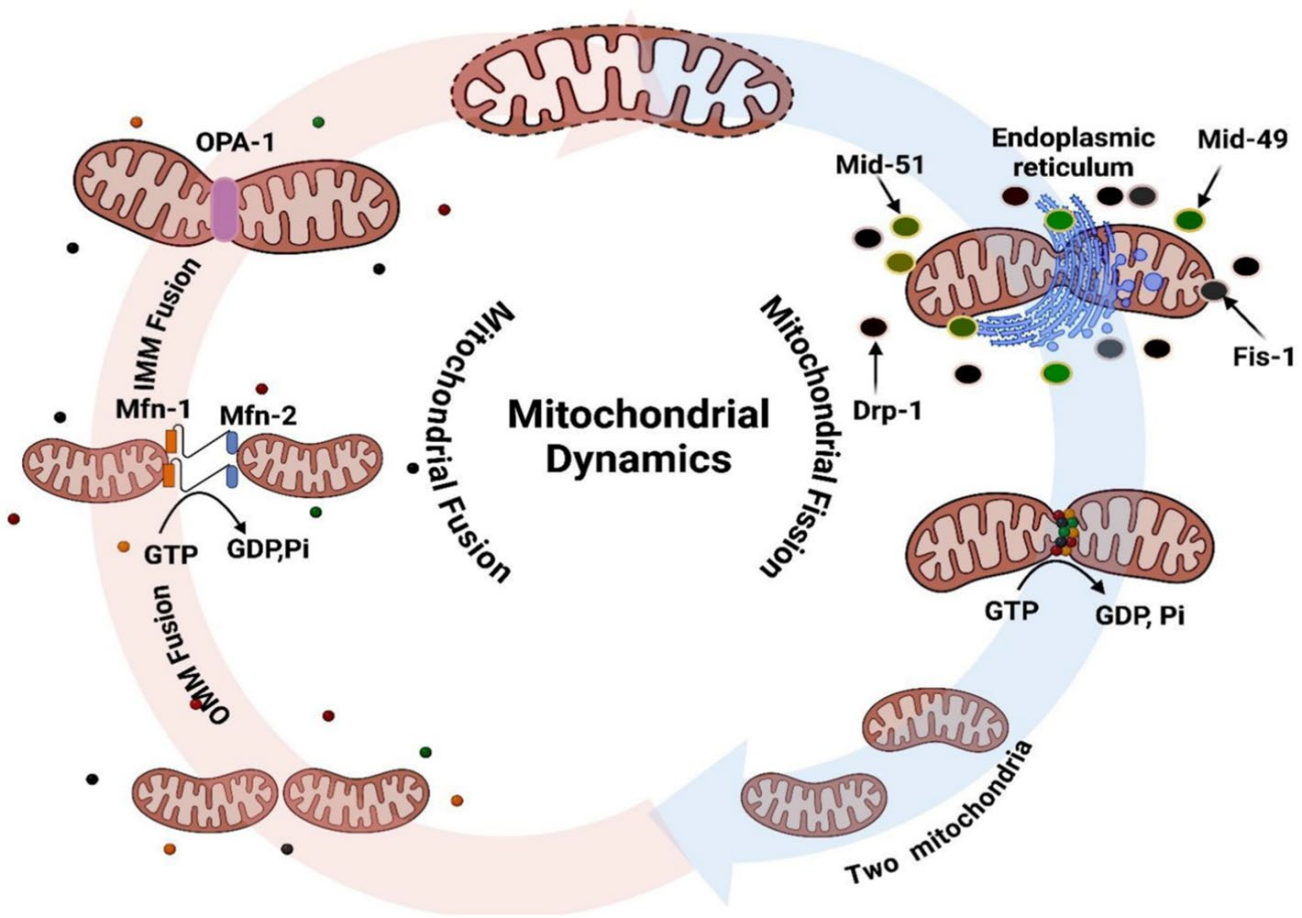
Mitochondrial fusion and fission

Dynamin-related protein 1 (Drp1) is the primary mediator of the phenomenon known as mitochondrial fission (Mito fission), which is the division of a single mitochondrion into two daughter mitochondria. Fission mitochondrial 1 (Fis1), mitochondrial dynamics protein of 49 kDa (MID49), and mitochondrial dynamics protein of 51 kDa (MID51) are mitochondrial receptor proteins that help recruit Drp1 from the cytoplasm to the mitochondrial membrane. Drp1 is a member of the GTP-binding proteins [51]. The mitochondrial membrane's Drp1 can then create a ring-like structure that encircles the mitochondrion and causes the membrane to rupture, necessitating GTP hydrolysis [52]. By altering Drp-1 stability and recruitment, phosphorylation of Drp1 can control Mito fission. In mitotic cells, phosphorylation of Drp1 on Ser585 stimulates mitosis, but phosphorylation of Drp1 on Ser637 can prevent mitosis (Fig. 11) [53].

The process of creating an interconnected mitochondrion from two parental mitochondria is known as mitochondrial fusion (Mito-fusion), and it can be separated into two types: inner mitochondrial membrane (IMM) fusion, which is mediated by optic atrophy 1 (OPA1), and outer mitochondrial membrane (OMM) fusion, which is mediated by mitofusin 1 (Mfn1) and mitofusin 2 (Mfn2) [54]. Dynamin-related GTPases include Mfn and OPA1. Interaction between Mfn1 and Mfn2 can mediate fusion between neighboring OMM. IMM fusion is mediated by OPA1 (Fig. 11) [55].

### Mitochondrial dynamics’ role in cancer

The development and spread of tumors are intimately linked to mitochondrial dynamics. The cancer adaptation is the change in mitochondrial dynamics brought on by changes in the environment surrounding a cell. Numerous malignancies have abnormalities in the dynamics of their mitochondria. Numerous cancer types exhibit up-regulation of proteins linked to fission and down-regulation of proteins linked to fusion [56].

Elevated DRP-1 activity cause excess mitochondrial fragmentation leads to a reduced uptake of Ca^2+^ by mitochondria, via activating the anti-apoptotic gene Bcl-_2_ by direct interaction of its BH4 domain with the Ca^2+^ release receptor (IP3R), that localized on the endoplasmic reticulum [ER] surface to control ER/Ca^2+^ content, thereby preventing the occurrence of excessive proapoptotic Ca^2+^ signals and mitochondrial Ca^2+^ overload, and this subsequently downregulates pro-apoptotic factors BAX and sustain the mitochondrial membrane potential.

**Fig. 12:**
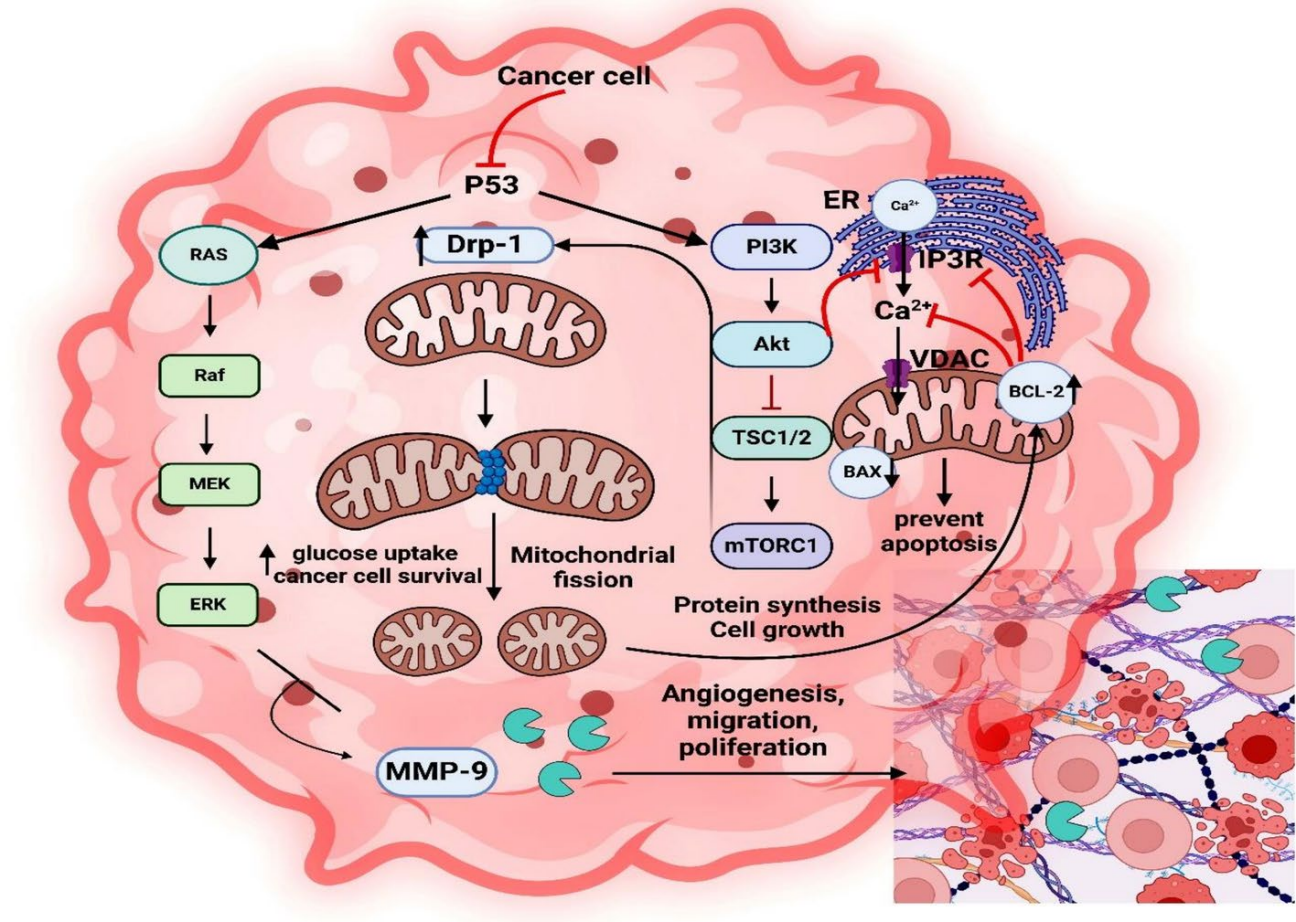
The role of mitochondrial dynamics in promoting cancer

Ras-Raf, p53-mediated mTORC1, and PI3K/AKT are examples of oncogenic signaling pathways that affect the dynamics of the mitochondria in cancer cells. Individually, Ras-Raf and PI3K/AKT stimulate mitochondrial fission, which raises glucose absorption and lowers OXPHOS, causing a metabolic shift to glycolysis. AKT has also been demonstrated to phosphorylate IP3R, which lowers ER/Ca^2+^ release and prevents cell death. As a result, the cell cycle accelerated, which increases the rate of mitochondrial DNA alterations and helps cancer cells survive. Moreover, mitochondrial fission encourages cytoskeleton reorganization, which improves the cancer cells' capacity for migration and proliferation. Additionally, blocking p53 increases the mTORC1 pathway, which phosphorylates Drp1 and increases ERK/MMP9 activity, making cancer cells more invasive (Fig. 12) [57].

### Therapeutic strategies based on mitochondrial dynamic-related factors

**Fig. 13:**
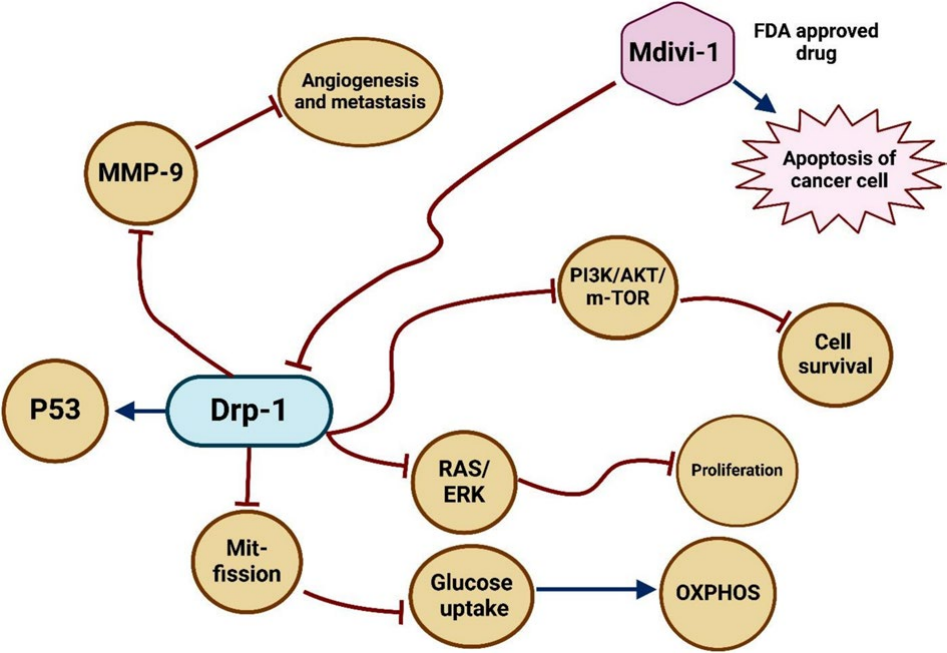
Therapeutic strategy based on mitochondrial dynamics-related factor

Targeting strategies related to mitochondrial dynamics is emerging as a viable cancer therapy approach. For example, the most effective medication for describing mitochondrial dynamics is Mdivi-1 [58]. It has some cytotoxicity to healthy cells, however, it can cause tumor cells to stop growing and undergo apoptosis [59]. Finding novel target molecules that prevent Drp-1 from mito-fission without harming healthy cells is therefore highly desired. Hekal et al. [60] clarified how novel α-aminophosphonates containing pyridine block DRP-1 mitochondrial fission following the prior need. Additionally, aberrant DRP1 phosphorylation brought on by p53 activation suppressing mTORC1 results in intermediate mitochondrial fusion, which lowers the production of ERK1/2 and MMP9 and stops cell migration. Thus, mitochondrial dynamics mechanistic pathways can be logically employed as a possible target for cancer treatment (Fig. 13) [61].

## Horizontal mitochondrial transfer (HMT) role in tumor progression

The involvement of mitochondria and mitochondrial DNA (mtDNA) in the development and advancement of cancer is now well-established. Moreover, several studies indicate that horizontal mitochondrial transfer (HMT) between cells is a crucial physiological process that facilitates intercellular communication. HMT is now recognized to be prevalent not just in normal tissues throughout the body, in response to stress and in benign illnesses, but also in tumor biology [62]. Initially, it was demonstrated that ρ0 tumor cells, which are devoid of mtDNA, exhibited slower division rates but may recover respiratory function by acquiring intact mitochondria and accompanying mtDNA from adjacent cells, thus facilitating accelerated cell division and reinstating their tumorigenic capacity. The mechanisms by which exogenous mitochondria drive cancer cell proliferation may relate to the generation of reactive oxygen species that stimulate delivery of mitochondria to cancer cells and that lead to activation of mitogenic signaling pathways, such as extracellular signal-regulated kinases. The function of mitochondrial transfer in cancer is intricate, as this mechanism has been documented to enhance neoplastic cell proliferation, invasiveness, immune evasion, and therapeutic resistance. Cancer cells are known to obtain mitochondria from other cells; however, the mechanisms of transfer and functional implications may depend on the cell of origin and recipient cancer cell type [63].

### Mechanisms of mitochondria transfer

Numerous unique pathways of intercellular mitochondrial transfer have been documented in the literature, with varying degrees of exploration among them. The transfer mechanisms can be classified into three categories: (A) establishment of temporary cellular linkages facilitating mitochondrial transfer across cells, (B) expulsion of mitochondria within extracellular vesicles for transport to target cells, and (C) liberation of free mitochondria for uptake by recipient cells [63].

#### Transient cellular connections

**Fig. 14:**
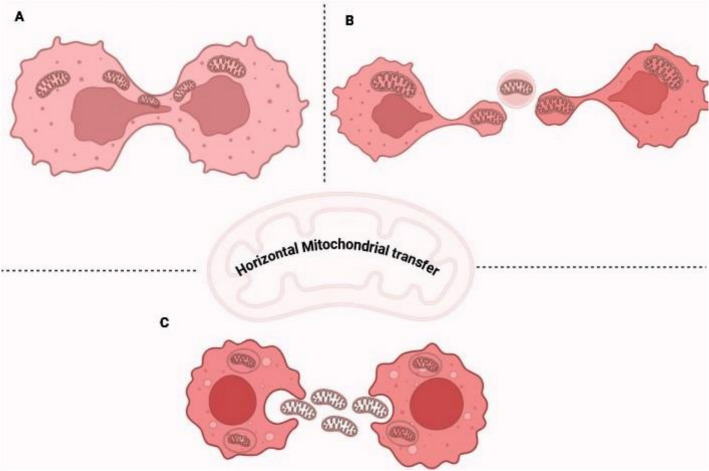
Horizontal mitochondrial transfer mechanism's in tumor progression

The predominant mechanism revealed is a cell contact-dependent process that entails the development of transitory intercellular connections termed tunnelling nanotubes (TNTs) and/or connexin 43 (Cx43)-mediated gap junctional channels (GJCs). Tunnelling nanotubes serve as the primary conduit for mitochondrial transfer among tumor cells. TNTs are nano-scale membranous conduits connecting cells, exhibiting diameters between 50 and 1500 nm and lengths of 5 to 200 μm, with certain TNTs attaining thicknesses of up to 700 nm. TNTs are not hollow membranous structures; rather, they are packed with cytoskeletal filaments. F-actin is prevalent in the majority of TNTs and serves as a vital structural element. The cross-linking of F-actin provides stiffness to the TNT, so stabilizing its outward expansion; concurrently, it facilitates the manufacture of TNTs and enables the movement of mitochondria along the cytoskeletal structures within TNTs [64]. These structures facilitate the bidirectional interchange of cytosolic and plasma membrane components, so forming a 'parabiotic' interaction between the involved cells. The development of TNTs is contingent upon growth-associated protein 43 (GAP43) and potentially Cx43, whereas mitochondria are transported along an actin-microtubule pathway via the Rho-GTPase Miro1 to infiltrate the cytoplasm of the receiving cell. The mechanism entails Miro1, was in conjunction with auxiliary proteins Miro2, transport associated protein 1 (TRAK1), TRAK2, and myosin XIX (Myo19), facilitating the binding of mitochondria to proteins directed by Kruppel-like factor 5 (KLF5). When bound, they form a motor-adaptor complex, thereby facilitating the transport of mitochondria within TNTs and regulating their movement [65]** (**Fig. 14A).

#### Extracellular vesicle-associated mitochondria

Another commonly reported mechanism of intercellular mitochondrial transport is the release and subsequent uptake of extracellular vesicle-containing mitochondria (EVMs) as cargo. EVMs can be classified according to their dimensions and mitochondrial content. Small extracellular vesicles, around 100–200 nm in diameter, frequently harbour oxidatively damaged mitochondrial components, as demonstrated for EVMs released by white and brown adipocytes. These extracellular vesicles are characterized by the presence of the tetraspanins CD63, CD9, and CD81. These structures are formed in a PINK1-dependent manner and are packaged into extracellular vesicles approximately 100 nm in size before being ejected from the cell, probably via multivesicular bodies. However, this ejection process is impaired without parkin, an E3-ubiquitin ligase that binds to PINK1 and labels damaged mitochondrial components to target them for degradation via mitophagy [66]. Numerous studies suggest that intact, functional mitochondria can be expelled from cells within around 1-μm-sized extracellular vesicles, which may also originate from the multivesicular body or bud off from the plasma membrane. Platelets release respiratory-competent mitochondria in extracellular vesicles following thrombin activation, a process seemingly facilitated by a plasma membrane budding mechanism rather than multivesicular body packaging [67]. These investigations suggest that mitochondria transfer by EVMs is not solely employed for the quality control of the donor cell but also influences the functioning of the receiving cell **(**Fig. 14B).

#### Release and capture of free mitochondria

The third mechanism of intercellular mitochondrial transfer involves the release of free or naked mitochondria, which are subsequently assimilated by recipient cells. This type of extracellular mitochondria was, to our knowledge, initially discovered in the blood of mice and humans, with activated platelets serving as one source, releasing both EVMs and free mitochondria in an estimated 2:1 ratio upon activation. Free mitochondria in the blood, devoid of an extracellular vesicle, measure roughly 0.5–1 μm in diameter and encompass a complete mtDNA genome. Numerous sources of circulating mitochondria likely exist. Cell fusion can facilitate the transfer of mitochondria between cells. This process transpires by both partial cell fusion mediated by tunnelling nanotubes (TNTs) and complete cell fusion reliant on mitochondrial fission proteins, including dynamin-related protein 1 (DRP1) and mitochondrial fission 1 protein (FIS1). The liberated mitochondria are then engulfed through a phagocytic process, presumably macropinocytosis, or assimilated by recipient cells in a heparan sulphate (HS)-dependent fashion. Certain naked mitochondria may evade the endosomal compartment subsequent to their entrapment [68] (Fig. 14 C).

### HMT and its therapeutic strategy

There were various techniques to impede mitochondrial transport and maybe improve the effectiveness of cancer treatment. One strategy involves targeting microtubule polymerization, essential for the creation of tunnelling nanotubes (TNTs) that enable mitochondrial transport. Taxanes and Vinca alkaloids, prevalent chemotherapeutic drugs, have demonstrated partial inhibition of mitochondrial transport by obstructing microtubule polymerization [69]. Consequently, these medicines may serve as adjuvant therapies to inhibit or diminish the transfer of mitochondria from resistant to vulnerable cancer cells. Moreover, M-sec, a marker and regulator of TNT production, has been suggested as a potential targetable inhibitor of mitochondrial transfer [70]. Inhibiting M-sec expression or function may obstruct TNT production and diminish mitochondrial transport among cancer cells. Other molecules implicated in TNT production and mitochondrial fission, including Rho GTPases, may potentially be considered for therapeutic targeting [71].

## Metabolic reprogramming cancer signaling pathways

Significant metabolic changes in cancer cells enable their quick development, survival, and capacity to adapt despite several harsh conditions. Here, we highlight the vital aspects of cancer metabolic reprogramming [72].

### Dysregulation of glucose metabolism

The critical aspect in altering cancer cells' metabolism is glycolysis, the primary metabolic process that turns glucose into pyruvate [73]. In healthy cells, OXPHOS in the mitochondria under aerobic conditions usually follows glycolysis, producing up to 36 ATP molecules for every glucose molecule. On the other hand, glycolysis in cancer cells only generates two ATP molecules for every glucose molecule. This inefficiency was addressed by upregulating glucose transporters, specifically GLUT1, and glycolytic enzymes, such as hexokinase [HK], which convert glucose into glucose-6-phosphate (G6P) [74].

**Fig. 15:**
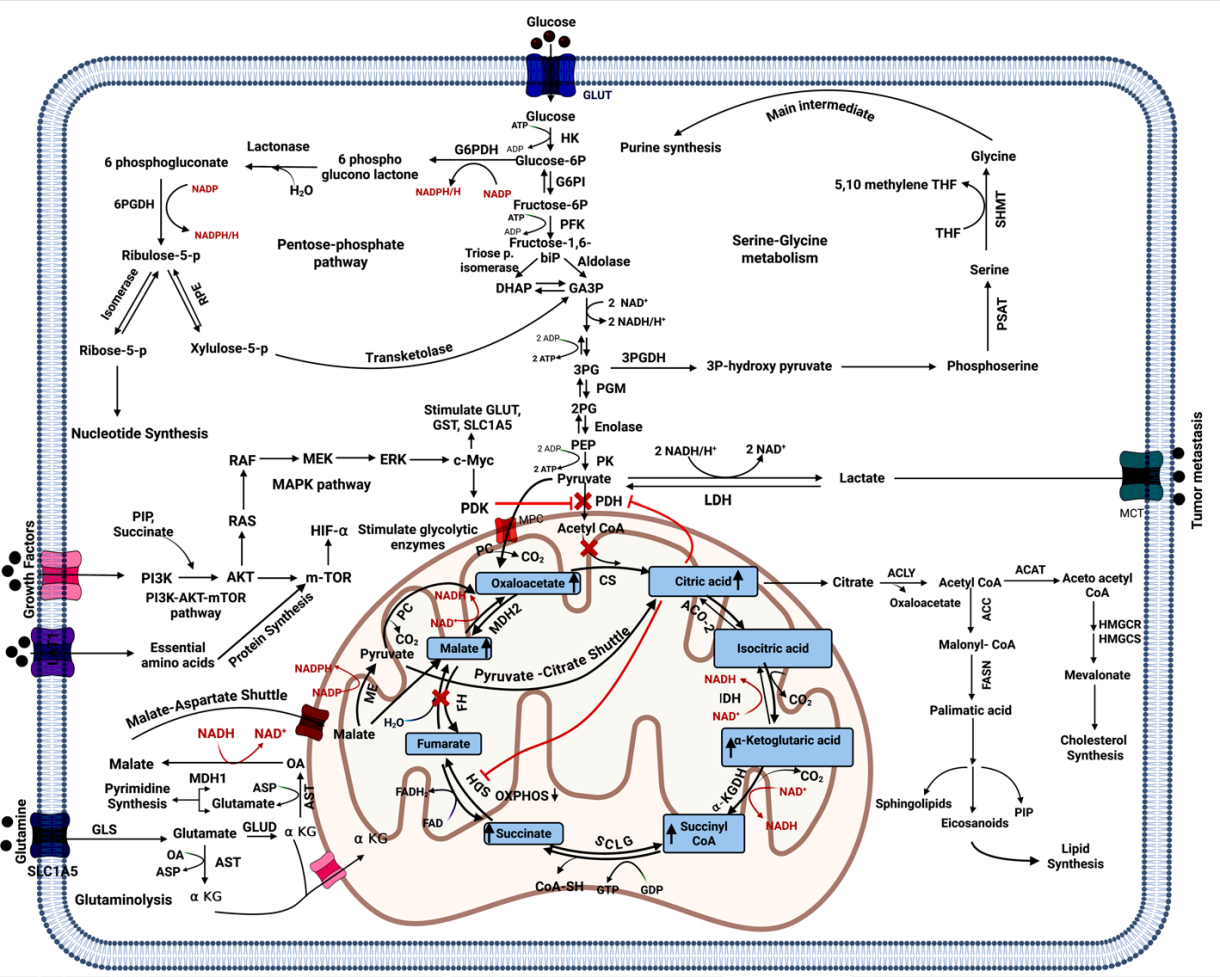
Crosstalk of cancer metabolic pathways

Additional important glycolytic pathway regulating enzymes, including pyruvate kinase (PK), which changes phosphoenolpyruvate into pyruvate, and phosphofructokinase (PFK), which changes fructose-6-phosphate (F6P) into fructose1-6 bis phosphate (F1,6BP), are elevated in cancer cells, resulting in increased glycolytic flow [75]. Instead of oxidizing pyruvate in the mitochondria, cancer cells preferentially use lactate dehydrogenase A (LDHA) to convert it to lactate, even when oxygen is present. The monocarboxylate transporter (MCT) is overexpressed and permits the outflow of large volumes of lactate generated by oxygen-depleted cells; this phenomenon is called the Warburg effect (Fig. 15) [76].

In many malignancies, the pentose phosphate pathway (PPP) is upregulated in conjunction with glycolytic turnover because of the increased energy demand of cancer cells for proliferation [77]. PPP supplies NADPH, which is necessary for the production and survival of fatty acids, as well as pentose phosphate, which is specifically needed for nucleic acid synthesis [78]. The enzyme that connects glycolysis and the PPP pathway is glucose-6-phosphate dehydrogenase (G6PD), which converts glucose-6-phosphate (G6P) into 6-phosphogluconate (6-PG). Additionally, the primary regulating enzyme in PPP is 6-phosphogluconate hydrogenase (6-PGD), which transforms 6-PG into ribulose-5-phosphate (Ru5P). Transketolase, a precursor to the glycolytic pathway flow, transforms 6-PG into glyceraldehyde 3-phosphate (Fig. 15) [79].

Furthermore, Hexokinase inhibits the attachment of BAX to the mitochondrial membrane, which mimics the occurrence of apoptosis by preventing the formation of a gap in the outer membrane of the mitochondria and the outflow of cytochrome C [80].

It is known that tumor suppressors like p53 work to inhibit glycolysis, whereas some oncogenes, including HIF-1α, c-Myc, and AKT, stimulate it. The glycolytic phenotype is frequently promoted by p53 depletion in cancer cells [81]. On the other hand, HIF-1α is a key regulator of the physiological response to hypoxia and stimulates glycolysis in conjunction with c-Myc through several mechanisms: it upregulates almost all glycolytic enzymes, such as pyruvate kinase (PK), phosphofructokinases (PFK), and glucose transporters GLUT1, which improves glucose uptake; it also increases the LDHA and MCT expression which facilitates lactate production and export. HIF-1α also inhibits mitochondrial biogenesis and oxidative phosphorylation. [82].

Although the Warburg phenomenon is essential to the metabolism of cancer cells, it is not the only metabolic change seen in these cells. Many additional metabolic pathways undergo reprogramming to accommodate the distinct requirements of tumor growth and survival. Glycolysis is also increased by signaling pathway activation such as PI3K/AKT/mTOR, which aids in the metabolic reprogramming seen in cancer cells. AKT may affect mitochondrial activity to maintain TCA cycle flux and control metabolism by activating mTORC1 [83, 84]. To take advantage of the metabolic weaknesses of cancer cells, targeting glycolysis presents a viable approach to cancer treatment.

### TCA cycle alterations

Mitochondria is the host to the TCA cycle, which oxidizes acetyl-CoA from proteins, lipids, and carbohydrates to produce ATP, NADH, and FADH2 [85]. The electron transport chain and oxidative phosphorylation depend on these products. To ensure cancer cell survival, several alternate strategies were mediated to maintain an active Krebs cycle if the Warburg effect depletes the intermediates of the TCA cycle needed to support vital biosynthesis pathways or inactivate essential enzymes in the cycle [86]. Furthermore, accumulating evidence now suggests that cancer cells are metabolically flexible, modifying their metabolic patterns according to their location and the nutrients available in their surroundings [87].

#### Citrate

The first TCA cycle intermediate is citrate, which is created when citrate synthase (CS) condenses oxaloacetate [OA] and acetyl-CoA. The movement of citrate between the cytosol and the mitochondrial matrix is mediated by the mitochondrial citrate transporter, SLC25A1. Furthermore, the SLC13A5 receptor is linked to the plasma membrane and speeds up the cancer cells' absorption of blood's citrate. Cancer migration and invasion are facilitated by citrate accumulation because cytosolic citrate is a precursor molecule for lipid production. Also, aconitase [ACO] controls the conversion between citrate and aconitate in the mitochondria (Fig. 15) [88].

Cancer cells can alternate the TCA cycle's flow to mediate citrate's availability for lipid synthesis. Where, cytosolic ATP-citrate lyase (ACLY) transforms citrate into acetyl-CoA and oxaloacetate. Afterward, acetyl-CoA is channeled into the synthesis of palmitate and other fatty acids required for membrane formation during cell proliferation, which also induces some inflammatory mediators like prostaglandin (PG) and nitric oxide. Moreover, citrate acts as an allosteric inhibitor to phosphofructokinase 1 (PFK1), the main step enzyme in glycolysis. But cancer cells circumvent this discharge in glycolysis by activating the formation of glyceraldehyde -3- phosphate from ribulose-5p and xylulose-5p key compounds in PPP via transketolase enzyme to still shifting towards increase production of lactate (Fig. 15) [89].

Citric acid stops pyruvate dehydrogenase (PDH) from producing acetyl-CoA from glycolysis. OA produced by ACLY's citrate conversion can also be utilized to produce aspartate, a metabolite that promotes the growth of cancer [90]. It is also known that citrate inhibits succinate dehydrogenase in the TCA cycle, which stops fumarate from forming and, consequently, malate from forming by decreasing fumarate hydratase activity and lowering OXPHOS [91]. Citrate's function has been summed up as suppressing catabolic processes linked to energy production and increasing lipogenesis, which is necessary to provide the membrane lipids required by a rapidly proliferating cell (Fig. 15) [92].

#### α-Ketoglutarate

A crucial metabolite of the TCA cycle, α-Ketoglutarate (α-KG), also known as 2-oxoglutarate, is produced when the isocitrate is oxidatively decarboxylated by isocitrate dehydrogenase [IDH]. The cell contains three different types of IDH: IDH1 is in the cytoplasm, IDH2 and IDH3 are found in mitochondria. Glutamate metabolism through anaplerosis can provide α-KG to power the TCA cycle, which supplies citrate and OA, which are used for lipid and amino acid synthesis when cataplerotic depletion occurs [92]. Glutamate dehydrogenase (GLUD; also known as GDH) and glutaminase (GLS) perform two series of deamination processes to convert glutamine into α-KG and glutamate, respectively. Glutamate–oxaloacetate transaminase (GOT) in the mitochondrion catalyzes the process of transamination OA to produce α-KG from glutamate, which also produces aspartate (Fig. 15) [92].

Additionally, α-KG is an essential nitrogen source for cancer metabolic pathways. The Warburg effect states that lactate is produced by cancer cells channeling glucose carbons. This is problematic because it deprives the cells of the pyruvate that is produced from glucose and used to power the TCA cycle. Surprisingly, citrate plays a significant role in the de novo lipid synthesis of rapidly growing cells. [93]. Therefore, glutaminolysis is necessary for the TCA cycle to mediate cancer cells engaging in aerobic glycolysis. Furthermore, to enhance the reductive carboxylation of α-KG, Hypoxia-inducible factor lowers citrate levels in proportion to α-KG and also encourages the Warburg effect. Glutaminolysis provides α-KG, which increases mTORC1 activity and helps cells survive in situations with low levels of amino acids (Fig. 15) [94].

#### Succinyl-CoA

The TCA cycle’s four-carbon phase begins with the production of succinyl-CoA from α-KG via oxidative decarboxylation using α-ketoglutarate dehydrogenase (α-KGDH). Further, succinyl-CoA synthetase catalyzes the transformation of succinate from succinyl-CoA. Additionally, this reaction is linked to ATP production without oxygen via substrate-level phosphorylation (SLP), which makes it a potential energy source in situations where aerobic glycolysis and OXPHOS energy production are possibly impaired (Fig. 15) [95].

#### Succinate

In many malignancies, the accumulation of citrate inhibits succinate dehydrogenase (SDH), which catalyzes the conversion of succinate to fumarate [96], resulting in succinate accumulation. By increasing anaerobic glycolysis, this accumulation enables succinate to bind to the succinate receptor (SUCNR1) on cancer cells, facilitating tumor spread and metastasis in a manner reliant on the AKT/mTOR/HIF-1α signaling axis (Fig. 15) [72].

#### Malate and oxaloacetate

Malate dehydrogenase 1 (MDH1) and 2 (MDH2) are commonly activated in malignancies, according to several studies [97]. MDH1, which is found in the cytosol, facilitates OA's conversion to malate. Malate then enters the TCA cycle via the malate transporter, and MDH2 transforms it back into OA. This is known as the "malate-aspartate shuttle" because OA then transaminated into aspartate and αKG, the primary intermediate in the Krebs cycle. It has been demonstrated that MDH1 aids lactate dehydrogenase in regenerating NAD^+^, a cofactor for glycolysis [98]. Moreover, malic enzyme 1 (ME1) converts malate to pyruvate, and decarboxylase converts pyruvate to OA [99]. Furthermore, a process known as the "pyruvate-citrate shuttle" occurred when OA and acetyl-CoA, which are created by ATP-citrate lyase, reacted to produce citrate via citrate synthase (Fig. 15) [100]. These findings lend support to the theory that malate buildup is required for cancer progression via increasing glycolytic flow. Cancer therapy can greatly benefit from targeting the TCA cycle.

### Amino acids metabolism dysregulations

Amino acid metabolism is inextricably linked to the glycolytic pathway, in which amino acid pools can produce multiple Krebs cycle components via paths of anaplerotic, as well as further metabolites like glucose, purine, lipids, and pyrimidine precursors [101]. During glucose deprivation, cancer cells rely on their amino acid pools for energy. The primary amino acid needed for cancer growth is glutamine, which gives cancer cells the carbon and nitrogen they need for biosynthesis and cellular homeostasis [102]. Glutaminolysis transforms glutamine into α-ketoglutarate and glutamate, which can be utilized in the TCA cycle to support metabolic processes and produce energy. This route is especially significant in cancer metabolism because many cancer cells are highly dependent on glutamine, which serves as a key fuel source for survival and proliferation [103].

Glutaminolysis starts when the body's abundant amino acid glutamine enters the cell through the neutral amino acid transporter (LAT1) and alanine-serine-cysteine transporter 2 (ASCT2). ASCT2 is a sodium-dependent transmembrane transporter that carries glutamine and other neutral amino acids across the plasma membrane. ASCT2 is encoded by the SLC1A5 gene [104]. The enzyme glutaminase (GLS) transforms glutamine into glutamate once it is inside. Glutamate dehydrogenase (GDH) or transaminases can then further convert glutamate to α-ketoglutarate. Aspartate, which is produced by the transamination of oxaloacetate, is utilized in the purine and pyrimidine base biosynthesis (Fig. 15) [105].

Another essential amino acid for cancer cells is serine, which participates in methyl transfer processes and indirectly contributes to the creation of purine and pyrimidine bases. Three events result in the production of serine from 3-phosphoglycerate, a crucial step in the glycolytic pathway. In the initial reaction, 3 phosphoglycerate undergoes a transformation into 3 phosphohydroxypyruvate by means of 3 phosphoglycerate dehydrogenases. Following transamination, 3 phosphohydroxypyruvate is changed into 3 phosphoserines. Phosphoserine phosphatase separates phosphate in the final step, converting 3-phosphoserine to serine [106]. Under the action of hydroxymethyl transferase, serine transfers one methylene agent onto tetrahydrofolate (THF), producing glycine and methylene THF (Fig. 15) [107].

The AKT/PI3K signaling system appears to exert many of its effects on amino acid and nitrogenated organic base metabolism through mTORC1. mTORC1 stimulates glutamine uptake, glutaminase, and glutamate dehydrogenase, leading to improved glutamine conversion and α-ketoglutarate synthesis. mTORC1 appears to stimulate the manufacture of purine bases by upregulating the activating transcription factor 4 (ATF4) and enhancing the THF cycle [108]. mTORC1 stimulates the trifunctional and crucial enzyme CAD (carbamoyl-phosphate synthetase 2, aspartate trans-carbamylase, and dihydroorotase), which is involved in the manufacture of pyrimidines. Additionally, c-Myc affects the metabolism of amino acids by increasing the expression of glutaminase and glutamine transporters. [109]. Furthermore, cancer also overexpresses LAT1, which is the main transporter of branched-chain amino acids (BCAAs) that the body cannot synthesize. This transporter provides the nutrients required for tumor growth (Fig. 15).

### Lipid metabolism alterations

Lipid metabolism is significantly altered in cancer cells as opposed to healthy cells. These modifications include altered fatty acid oxidation, improved fatty acid absorption, and greater de novo lipogenesis [110]. Many malignancies have an enhanced de novo lipogenesis process, which is the mechanism by which cells create fatty acids from non-lipid precursors. ACLY, acetyl-CoA carboxylase [111], and fatty acid synthase (FASN) are important enzymes in this pathway that are frequently overexpressed in tumor cells (Fig. 14). Fatty acids that are necessary for energy production, membrane biogenesis, and the creation of signaling molecules are thus abundantly available. The overexpression of fatty acid transport proteins (FATPs), receptor contributes to the increased fatty acid uptake in cancer cells, which supply building blocks for membrane production and an extra source of energy [112].

Cancer cells generate energy through fatty acid oxidation (FAO) using free fatty acids when they are malnourished. In cancer cells, the c-Myc oncogene frequently reprogrammed these free fatty acids through FAO, which is carried out by carnitine palmitoyl transferase (CPT). ATP, NADPH, and acetyl-CoA are all essential for preserving the energy homeostasis of cells and are supplied by FAO [113]. Proliferating cancer cells have significant energy requirements, which are met by the ATP produced by FAO. Moreover, acetyl-CoA generated from FAO and cytosolic citrate is critical for the synthesis of fatty acids and cholesterol, as well as for the creation of membranes and signaling molecules. [114].

In cancer, lipids are essential signaling molecules in addition to being energy sources. Cell proliferation, survival, migration, and invasion are all significantly impacted by lipid-signaling pathways, which are mediated by substances including phosphoinositides, sphingolipids, and eicosanoids [115]. Phosphatidylinositol (3,4,5)-trisphosphate (PIP3) is a lipid-based messenger that attracts and phosphorylates PI3K, resulting in activating AKT to support cell survival and proliferation [116, 117]. Additionally, sphingolipid metabolism is closely related to the development of cancer [118] (Fig. 15).

### Crosstalk between metabolic pathways in cancer cells

Cancer cells use the interconnected metabolism of glucose, amino acids, lipids, and nucleotides to sustain their unrestricted growth and development. Cancer cells primarily use glucose as their energy source during the start phase. Also overexpressed the glycolytic enzymes HK, PFK, and LDH, thereby accelerating anaerobic glycolysis. They also limit aerobic glycolysis by upregulating PDK, which stops pyruvate from being converted to acetyl-CoA and drives it to create more lactate. [119].

P53, c-Myc, HIF-1, and PI3K/AKT/mTOR activation are the main causes of anerobic glycolysis in cancer. When glucose levels in the environment are low during the development of cancer, the cells employ other sources of carbon and nitrogen, such as amino acids, nucleotides, and lipids, to create intermediates of the TCA cycle and use OXPHOS as a source of energy. Cancer cells can, therefore, switch between OXPHOS and glycolysis simultaneously, depending on the situation, owing to their metabolic flexibility [120].

In nutrient-deficient conditions, cancer cells employ lipids and amino acids as secondary energy sources. BCAAs and glutamine, respectively, generate the TCA cycle intermediates pyruvate and α-ketoglutarate, which in turn generate NADH and FADH2 and go via OXPHOS to generate ATP. Also, c-Myc activates the glutamine transporter SLC1A5 to enhance glutamine absorption from the surroundings and enhance GLS expression, which in turn triggers an increase in glutaminolysis [121].

The conversion of glucose-6-phosphate (a glycolytic intermediary) to 6-phosphogluconate and the synthesis of ribose-5-phosphate (purine synthesis substrate) are what drive the glucose to the PPP pathway's flux. Nucleotide metabolism is also linked between the metabolism of glutamine and PPP (ribose-5-phosphate), as both of which are necessary for DNA synthesis, which is responsible for cancer survival and growth. Under nutrient-rich circumstances, cancer cells use lipogenesis to create lipid molecules from acetyl-CoA produced from cytosolic citrate; during the mal-nutrient conditions, lipolysis breaks down lipid droplets to create free fatty acids, which subsequently undertake FAO to provide energy needed for cancer cell proliferation [114, 122].

When a metabolic pathway is impaired due to dietary deficiency or external stressors, cancer cells compensate by enhancing alternative metabolic pathways to sustain their growth and proliferation, owing to the interconnectedness of glucose, amino acid, lipid, and nucleotide metabolism [123]. Thus, focusing on a single metabolic pathway is not a viable therapeutic approach for treating cancer; rather, combination therapy should be prioritized since it simultaneously targets multiple metabolic pathways that are upregulated in cancer cells (Fig. 15).

### Therapeutic strategies targeting metabolic alteration in cancer cells

Targeting combined techniques represents a promising strategy for cancer treatment. This approach is more successful and could perhaps enhance outcomes for cancer patients.

#### Strategies using oncogenes and tumor suppressor genes

Cancer dysregulation for metabolic pathways is greatly impacted by the actions of tumor suppressors and oncogenes [124]. Targeting cancer treatments by blocking oncogenes and turning on tumor suppressor genes is essential.

##### Oncogenes

**Fig. 16:**
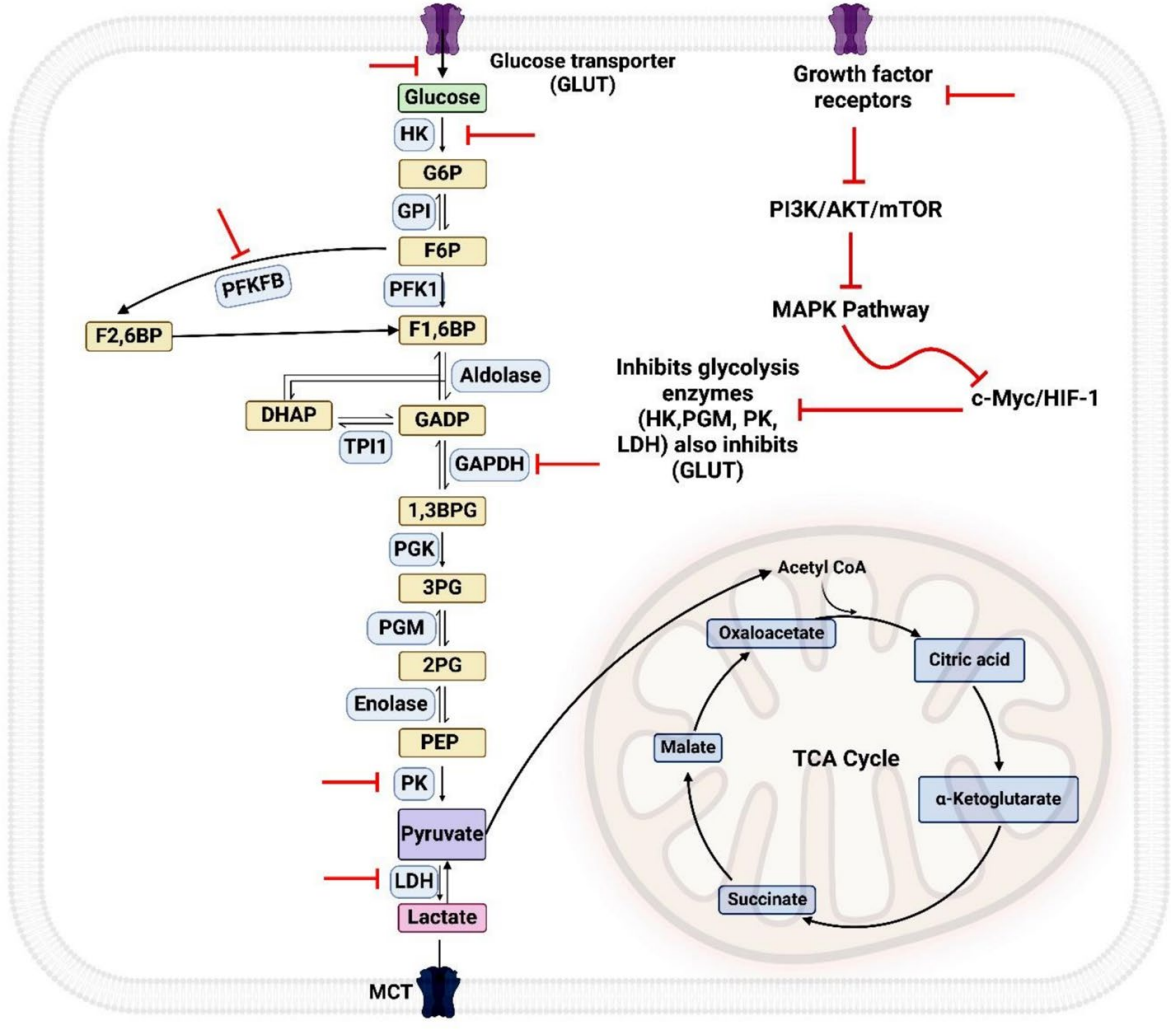
Targeted strategies to inhibit the Warburg effect (anaerobic glycolysis)

**Fig. 17:**
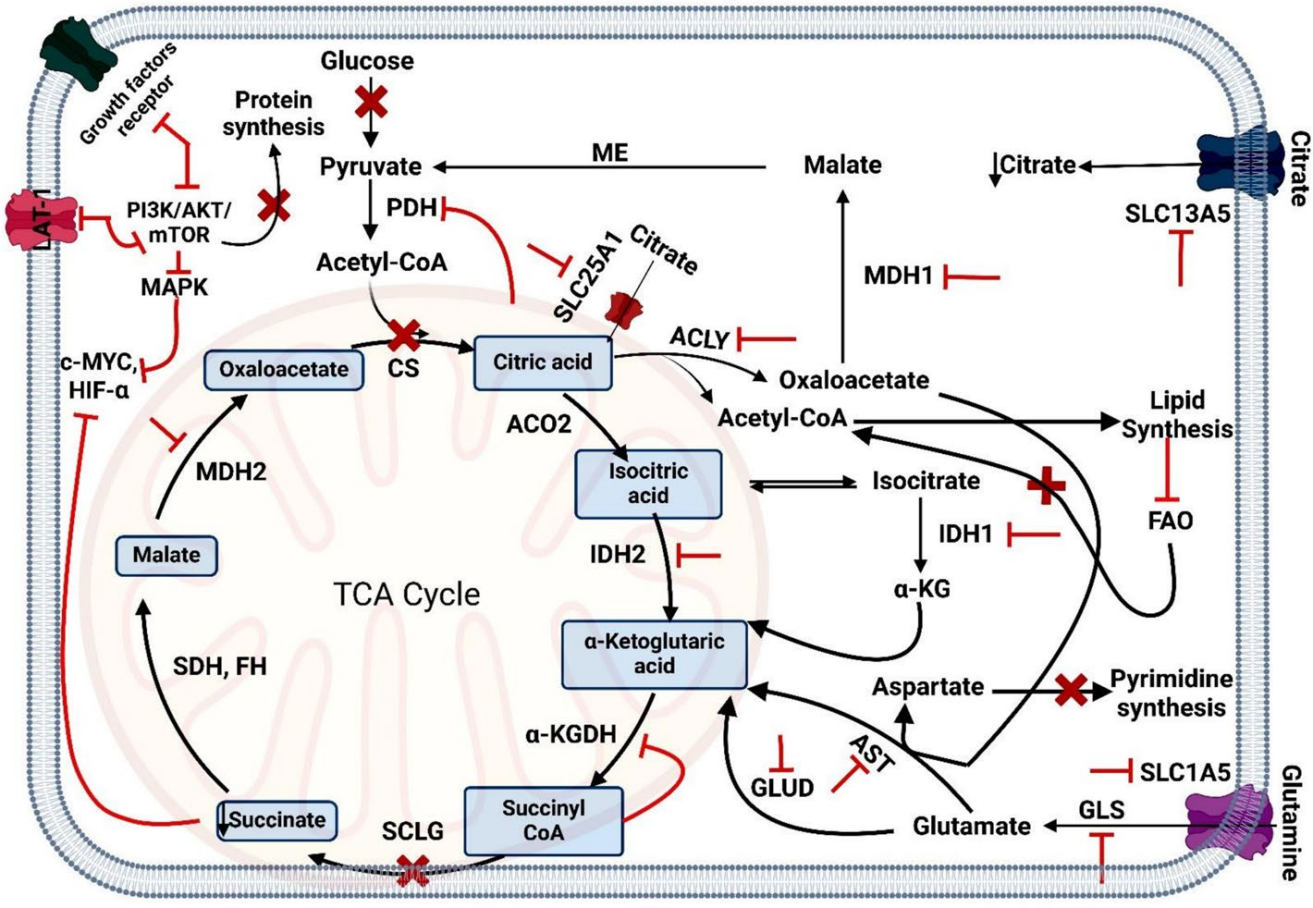
Interference of metabolic pathways for effective therapeutic strategy

Many oncogenes are recognized for modifying metabolic pathways to satisfy the energy and biosynthetic demands for rapidly growing cancer cells. c-Myc increases glutamine metabolism, which feeds into the TCA cycle and stimulates the glucose transporters and glycolytic enzymes [125]. Similarly, the RAS protein family initiates pathways such as MAPK, YAP/TEAD and PI3K/AKT that speed up the intake of glucose, glycolysis, and lipid synthesis, all of which are essential for cell growth and proliferation [126] (Figs. 16, 17).

Therefore, KRAS can be blocked by three mechanisms: obstructing ligand binding to the receptor tyrosine kinase, inhibiting SOS (son of sevenless) to prevent the conversion of GDP to GTP, and averting mutations at the 12th codon (G12) [127]. Various strategies have been employed to block its activity through small compound inhibitors, including SML-10-70-1 [128] and SML8-73-1 [129], based on the binding pockets present in KRAS mutants. Also, H-REV-107 is a peptide-based selective inhibitor that targets the nucleotide binding site of KRASG12 [130]. The small molecule inhibitors that non-covalently target the switch II pocket of KRAS include AMG510, ARS853, MRTX849, ARS1620, and GDC6036 [131]. MRTX849 (Adagrasib) is an FDA-approved covalent inhibitor, while AMG510 (Sotorasib) is a first-in-class, orally bioavailable covalent KRAS inhibitor that demonstrating antineoplastic efficacy in clinical trials. Furthermore, ARS853, GDC6036 (Divarasib), JDQ443, ARS1620, JNJ74699157, and LY3537982 have all received approval as inhibitors of KRAS, representing some of the most effective cancer treatment strategies [132]. A Phase 1 trial integrating RMC-6291 with RMC-6236 is presently under progress for solid tumours [133]. BI-2865 is a new pan-KRAS inhibitor [134]. ADT-007 is a novel pan-RAS inhibitor that obstructs GTP-binding activation and subsequent effector engagement by binding to nucleotide-free RAS [135]. MRTX1133 is currently in first clinical studies labeled in patients with advanced solid tumors harboring a KRAS protein [136].

Moreover, numerous resistance mechanisms to therapies targeting the PI3K/Akt/mTOR pathway have been identified, constraining their therapeutic effectiveness in cancer cells. Regarding PI3K inhibition, various mechanisms of pharmacological resistance have been documented. Initially, the participation of the PI3K/Akt pathway in glycolysis [137] indicates that PI3K inhibition leads to hyperglycemia, subsequently activating insulin signaling, particularly via the insulin-like growth factor 1 receptor (IGF1R). This signaling can subsequently reactivate the PI3K/Akt/mTOR pathway [138], activate the Janus kinase 2 (JAK2)/signal transducer and activator of transcription 5 (STAT5) pathway to enhance cell proliferation and survival [139], or activate the oncogenic kinase MAPK, which can independently stimulate the Akt/mTOR axis without PI3K involvement, thereby conferring resistance to PI3K inhibitors [140]. Furthermore, the overexpression of the pseudo receptor tyrosine kinase 7 (PTK7) often results in the rapid activation of the non-canonical Wingless-related integration (Wnt) pathway following PI3K inhibition [141].

The exploration of resistance mechanisms to therapies targeting the PI3K/Akt/mTOR pathway has led to the development of combination strategies with other targeted agents to enhance their efficacy [142]. The combination of gedatolisib with the ADC cofetuzumab pelidotin, which targets PTK7, was assessed in the phase I clinical trial NCT03243331. This treatment combination seeks to bypass the resistance mechanism induced by Wnt pathway activation via PTK7 inhibition [141]. Preclinical investigations have revealed a synergistic potential between PI3K inhibitors and PARP inhibitors. Consequently, a phase I study (NCT01623349) was initiated to evaluate the combination of the PI3K inhibitor buparlisib and the PARP inhibitor olaparib [143]. Preclinical investigations have shown a synergistic relationship between eribulin and everolimus or the PI3K inhibitor copanlisib in triple-negative breast cancer cell lines [144]. The dual combination of the PI3K inhibitor alpelisib and the ERK inhibitor trametinib inhibits cancer cell resistance [145]. PROTAC-based therapeutics (Proteolysis-Targeting Chimaeras) represent an innovative area in drug development research, specifically degrading components of the PI3K/AKT/mTOR pathway to avert drug resistance and reduce off-target damage [146]. These strategies offer potential for more resilient and efficacious cancer therapy alternatives.

Furthermore, The inhibition of the YAP/TAZ-TEAD transcriptional complex with new TEAD inhibitors has demonstrated promising outcomes for therapeutic intervention in cancer. The majority of TEAD small molecule inhibitors focus on the lipid binding pocket of TEAD, that affect TEAD-regulated transcription operate by altering the auto-palmitoylation of a hydrophobic pocket in TEADs, which often stabilizes them and facilitates their physical interaction with YAP and TAZ. The second mechanism of TEAD inhibitors (TEADi) identified thus far are those that attach to the surface of TEAD1-4, directly obstructing their capacity to connect with YAP [147]. Verteporfin, an FDA-approved medication utilized as a photosensitizer in photodynamic treatment for macular degeneration, was effectively repurposed as a YAP-TEAD inhibitor [148]. Compounds have been found to target the interactions between TEAD and YAP based on comprehensive knowledge of these interactions. The small molecules that bind to the palmitate hydrophobic pocket of TEAD include both non-covalent and covalent inhibitors: MGH-CP1 [149], VT-104 [150], MSC-4106 [151], TM2 [152], GNE-7883 [153], TED-347 [154], DC-TEADin-02 [155], DC-TEADin-03 [156], Myf-01-037 [157], a Kojic acid analogue [158], Myf-03-69 [111], MGH-CP1 [159], and K-975 [160]. These compounds possess acrylamide moieties that can form covalent bonds with the catalytic cysteine at the entrance of the palmitate pocket. BY03 [161] and trisubstituted pyrazoles [162] have been formulated as inhibitors of interface 2. The binding of the inhibitor to interface 3 encompasses CDP3.1 [163], which was discovered through a simulated screening process. Dioxo-benzo[d]isothiazole was recognized as an inhibitor of TEAD-binding ligands [164]. Additionally, the novel covalent and irreversible YAP/TAZ-TEAD inhibitors, SWTX-143 and SW-682, were examined [165, 166]. Pazopabin served as a dual function by inducing both proteasomal degradation and phosphorylation of YAP/TAZ [167]. IAG933 is an inhibitor of pan-TEAD protein–protein interactions [147]. A TEAD dominant-negative protein inhibitor (TEADiv2), physically derived from the YBD, significantly suppressed the transcriptional activity of TEAD [168].

Certain TEAD inhibitors have entered clinical trials and are presently being evaluated for their efficacy in metastatic malignant mesothelioma and other solid tumours. At present, other TEAD inhibitors were undergoing human clinical trials, including VT3989 (NCT04665206) [150], IAG933 (NCT04857372) [147], ISM6331 (NCT06566079) [169], SW-682 (NCT06251310) [166], ION537 (NCT04659096) [170], and BPI-460372 (NCT05789602) [171]. As TEAD inhibitors were a newly developing drug class, all current clinical trials are still in early phase trials, and many have not publicly reported their clinical findings at this time [172].

Also, HIF-1α stabilizes under hypoxia conditions in tumors, increasing the production of glycolytic enzymes and activating GLUT1 to promote Warburg phenomena. Moreover, HIF-1α upregulates VEGF, leading to increased angiogenesis of tumors [173]. These oncogenes change cellular metabolism, which boosts cancer cells' rapid growth and energy requirements (Figs. 16, 17**)**.

##### Tumor suppressor genes

Tumor suppressors, on the other hand, are genes that decrease cell development and proliferation to protect against cancer [174]. They frequently inhibit oncogene effects while also maintaining cellular homeostasis. In response to cellular stress and DNA damage, the tumor suppressor p53 induces cell cycle arrest and apoptotic death. p53 also controls metabolism by suppressing glycolysis and increasing OXPHOS [175]. Another important tumor suppressor is AMP-activated protein kinase (AMPK), which acts as a cellular energy sensor activated by low ATP levels. AMPK suppresses mTOR signaling and decreases protein synthesis [176].

#### Strategies pending through the main metabolic pathways

##### Glycolytic pathway

Targeting glycolysis represents a promising strategy for cancer therapy. The dependence of cancer cells on this pathway gives an urgent need for the development of several glycolytic inhibitors. GLUT1 inhibitors can reduce glucose uptake, depriving cancerous cells of their principal source of energy. Hexokinase inhibitors disturb the first steps of glycolysis and govern vascular formation, lowering glycolytic flux, affecting angiogenesis, and causing apoptosis [177, 178]. Furthermore, inhibitors of phosphofructokinase (PFKFB3) target a rate-limiting enzyme in glycolysis and angiogenesis, as VEGF triggers PFKFB3, which enhances endothelial cell migration through up-regulated HIF-1α and VEGFR2. [179].

Moreover, glyceraldehyde-3-phosphate dehydrogenase (GAPDH) inhibitors cause apoptosis by disrupting the glycolytic pathway and lowering ATP synthesis. A further tactic is to target pyruvate kinase, which is over-expressed in cancer cells [180]. A key step in glycolysis, the conversion of pyruvate to lactate, is prevented by LDH inhibitors. By blocking LDH, these substances interfere with glycolysis, lowering lactate production and preventing the growth of cancer cells [181]. Additionally, lactate inhibition may alter angiogenesis by blocking NF-κβ/IL-8 signaling [182]. Glycolytic inhibitors can also increase the effectiveness of other cancer treatments by making tumors more sensitive to traditional therapies like chemotherapy and radiation (Fig. 16).

##### TCA cycle

TCA cycle inhibitors as suppression of SLC25A1 impedes the transfer of citrate between the cytosol and mitochondrial matrix. Furthermore, the suppression of SLC13A5, which is anchored in the plasma membrane, mimics the blood's citrate uptake. Additionally, the production of citrate from OA and acetyl-CoA is prevented by inhibiting CS. IDH1/2 inhibition alters α-ketoglutarate formation. Furthermore, succinyl-CoA prevents succinate from forming by feedback inhibiting -ketoglutarate dehydrogenase. Furthermore, hangup mitochondrial malate and pyruvate transporters, malate dehydrogenase 1/2, diminishes the bypass of malate-aspartate and pyruvate-citrate shuttle systems [183, 184]. Combining TCA cycle inhibitors with chemotherapy or other treatments increases the effectiveness of treatment for a variety of cancer types (Fig. 17).

##### Glutaminolysis pathway

Many malignancies rely on glutaminolysis, which makes it a possible target for treatment. Inhibitors of glutaminase prevent glutamine from being converted to glutamate, which disrupts the TCA cycle's supply of α-ketoglutarate and lowers ATP and biosynthetic precursor production [185]. Furthermore, a more successful method of treating cancer might be achieved by merging glutaminase inhibitors with other metabolic inhibitors or traditional medicines. Targeting glutaminolysis and glycolysis, for example, can concurrently interfere with the two main metabolic processes in cancer cells, resulting in cell death (Fig. 17).

##### Amino acid transport

A potential strategy in cancer metabolism therapy is amino acid transport inhibition, which targets the vital nourishment that cancer cells depend on for survival and growth [186]. To meet their metabolic needs and rapid multiplication, cancer cells frequently show enhanced amino acid absorption. LAT1 and ASCT2 are two examples of transporters that are involved in amino acid uptake. By blocking these transporters, cancer cells can be effectively deprived of vital nutrients, which results in altered cellular metabolism and impaired protein synthesis and could eventually result in cell death [187]. Inhibiting amino acid transport can also increase metabolic vulnerabilities, making cancer cells more sensitive to other treatments. Amino acid transport inhibitors are, therefore, being investigated in conjunction with other therapeutic approaches to accomplish more efficient cancer management [188] (Fig. 17).

##### Lipid metabolism

Preclinical studies have demonstrated the inhibition of de novo lipogenesis enzymes as ACC and ACLY results in blocking FASN (fatty acid synthase) inhibits vascular sprouting and stops malonyl CoA from being converted to palmitic acid, both of which are being investigated as possible therapies [189]. Numerous enzymes in the FAO pathway are targeted by FAO inhibitors, such as 3-Ketoacyl-CoA thiolase [KAT], acyl-CoA dehydrogenase (ACAD), and carnitine palmitoyl transferase 1 (CPT1), which stop free fatty acid from being converted to acetyl-CoA [190]. Additionally, combining traditional treatments like chemotherapy with strategies to interfere with lipid-signaling pathways, like PI3K/AKT inhibition and sphingolipid metabolism modulators, may improve therapeutic efficacy [191] (Fig. 17).

## Data Availability

No datasets were generated or analysed during the current study.
